# Exosomal circular RNAs in tumor microenvironment: An emphasis on signaling pathways and clinical opportunities

**DOI:** 10.1002/mco2.70019

**Published:** 2024-11-24

**Authors:** Junshu Li, Wencheng Zhou, Huiling Wang, Meijuan Huang, Hongxin Deng

**Affiliations:** ^1^ Department of Biotherapy, Cancer Center and State Key Laboratory of Biotherapy West China Hospital, Sichuan University Chengdu China; ^2^ Department of Medical Aesthetics West China School of Public Health and West China Fourth Hospital Sichuan University Chengdu China; ^3^ Division of Thoracic Tumor Multimodality Treatment and Department of Medical Oncology Cancer Center West China Hospital, Sichuan University Chengdu China

**Keywords:** biomarker, circRNA, exosome, tumor microenvironment, tumor therapy

## Abstract

Exosomes can regulate the malignant progression of tumors by carrying a variety of genetic information and transmitting it to target cells. Recent studies indicate that exosomal circular RNAs (circRNAs) regulate multiple biological processes in carcinogenesis, such as tumor growth, metastasis, epithelial–mesenchymal transition, drug resistance, autophagy, metabolism, angiogenesis, and immune escape. In the tumor microenvironment (TME), exosomal circRNAs can be transferred among tumor cells, endothelial cells, cancer‐associated fibroblasts, immune cells, and microbiota, affecting tumor initiation and progression. Due to the high stability and widespread presence of exosomal circRNAs, they hold promise as biomarkers for tumor diagnosis and prognosis prediction in blood and urine. In addition, designing nanoparticles targeting exosomal circRNAs and utilizing exosomal circRNAs derived from immune cells or stem cells provide new strategies for cancer therapy. In this review, we examined the crucial role of exosomal circRNAs in regulating tumor‐related signaling pathways and summarized the transmission of exosomal circRNAs between various types of cells and their impact on the TME. Finally, our review highlights the potential of exosomal circRNAs as diagnostic and prognostic prediction biomarkers, as well as suggesting new strategies for clinical therapy.

## Graphical abstract

The exosomal circRNAs are differentially expressed in many types of tumors, including glioma, pituitary adenoma, esophageal squamous cell carcinoma, breast cancer, liver cancer, colorectal cancer, bladder cancer, osteosarcoma, and so on. And differentially expressed exosomal circRNAs affect tumor progression by regulating multiple target genes and signaling pathways, which have the potential to become targets for tumor diagnosis, prognosis prediction and tumor therapy.

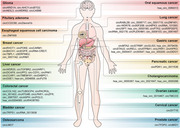

## 1 INTRODUCTION

Cancer is the most important social issue in current society.^1^ According to the 2022 cancer statistics, the number of new cancer cases is close to 20 million, and there are approximately 10 million new cancer deaths.^2^ Researchers predict based on population data that the number of new cancer cases will be 35 million per year by 2050, an increase of 77% compared with the level in 2020.^3^ In addition, the comprehensive incidence rate of men in multiple cancers is significantly higher than that of women in 2022, while there is no significant difference in mortality between men and women in cancer.^2^, ^4^, ^5^ In 2022, lung cancer was the most prevalent cancer, representing 12.4% of all global cancer cases. Breast cancer and colorectal cancer represent 11.6 and 9.6% of global cancer cases, respectively, while prostate cancer (PCa) and gastric cancer (GC) account for 7.3 and 4.9% of the total cancer cases.^2^, ^6^ Thus, investigating the molecular mechanisms of cancer is crucial for developing new clinical diagnostic and therapeutic strategies.

Exosomes are nanoscale extracellular vesicles (EVs) formed by endocytosis and can regulate disease progression by carrying various substances including lipids, proteins, DNA, mRNA, ncRNA (noncoding RNA, including miRNA, lncRNA, and circRNA) and metabolites. Proteins such as CD9, CD63, CD81, ALIX, HSP70, and TSG101 have been considered as biomarkers for EV detection.^7^ Genetic material is an important component of exosomes, with extensive research on noncoding RNA in exosomes. Compared with other types of noncoding RNA, the structure of circRNA is very unique. CircRNAs do not have a 3′‐terminal poly (A) tail and a 5′‐terminal structure, but rather a circular covalent closure formed by reverse and selective splicing of mRNA precursors. And the special structure of circRNA enables it to resist the degradation of exonucleases both inside and outside the cell, thus possessing high stability and exerting long‐lasting functions in the cell. In addition, circRNAs have a large number in exosomes and are not easily affected by external environments, which has attracted widespread attention.^8^ Currently, studies have found that multiple circRNAs play a crucial role in solid tumors (including lung cancer, esophageal cancer, GC, colorectal cancer, liver cancer, pancreatic cancer, renal cancer, bladder cancer, etc.) and have the potential to become therapeutic targets. For example, circSKA3 can maintain the stability of SLUG and activate the epithelial–mesenchymal transition (EMT) process in colorectal cancer by directly binding to SLUG in vivo and in vitro experiments. The use of ASOs (antisense oligonucleotides) to inhibit the level of circSKA3 can effectively weaken the interaction between circSKA3–SLUG and alleviate the metastasis of colorectal cancer.^9^, ^10^ In addition, circRNAs regulate key biological processes, including tumor proliferation, migration, apoptosis, autophagy, stemness, and drug resistance, which are crucial for tumor development and progression.^11^, ^12^, ^13^

Exosomal circRNAs mediate interactions between tumor cells and the tumor microenvironment (TME). The TME primarily consists of endothelial cells, cancer‐associated fibroblasts (CAFs), T cells, natural killer cells (NK cells), macrophages, and neutrophils.^14^, ^15^, ^16^ The interactions between various cells and signaling molecules in the TME affect the progression of cancer.^17^ Exosomes are usually released into the microenvironment in a paracrine manner and act on receptor cells, thereby transmitting genetic materials such as circRNAs between cells and regulating tumor angiogenesis, immune escape, and chemotherapy resistance.^18^, ^19^ CAFs, abundant stromal cells, communicate with tumor cells via exosomes to influence tumor progression in the microenvironment.^20^, ^21^ And a few exosomal circRNAs are absorbed by immune cells, and then regulate the immune escape process and affect the efficacy of immunotherapy. Exosomal circIGF2BP3 is notably increased in non‐small cell lung cancer (NSCLC) and impairs CD8^+^ T cell cytotoxicity by preventing programmed death ligand 1 (PD‐L1) ubiquitination.^22^ Moreover, a small portion of gut microbiota can regulate the biosynthesis of exosomal circRNAs in cells, which affects the development of tumors.^23^

CircRNAs in EVs hold potential as tumor diagnostic and predictive biomarkers, as well as therapeutic targets.^24^ Researchers identified significant differences in the expression of circ_0056285, circ_0007761, and circ_0047921 in serum exosomes of NSCLC patients compared with healthy individuals, aiding in the clinical diagnosis of NSCLC.^25^ In addition, EVs exhibit better biocompatibility in delivering nucleic acid substances compared with artificial nanoparticles.^26^, ^27^ Recent studies have found that exosomes derived from LoVo cells overexpressing circFNDC3B can inhibit angiogenesis and exhibit better antitumor effects in animal models of colorectal cancer.^28^ Another study utilized small interfering RNA (siRNA) targeting exosomal hsa_circ_0005963 to reverse oxaliplatin resistance by regulating the miR‐122/PKM2 axis and provide a new strategy for tumor therapy in colorectal cancer.^29^ Therefore, in‐depth research on new specific therapeutic approaches targeting circRNAs in vivo has demonstrated strong superiority.

In this review, we will summarize the downstream target genes and signaling pathways of exosomal circRNAs involved in key biological processes, including tumor growth, metastasis, apoptosis, autophagy, drug resistance, and immune escape. In addition, we will elucidate the molecular mechanisms of exosomal circRNA interactions within various cells of the TME, including tumor cells, tumor‐associated fibroblasts, immune cells, and gut microbiota. Finally, we will delve into the clinical application prospects of exosomal circRNAs in tumor diagnosis, patient prognosis prediction, drug efficacy prediction, and tumor therapy, providing promising new diagnostic and treatment strategies for cancer patients.

## 2 BIOGENESIS OF EXOSOMES AND EXOSOMAL circRNAs

EVs are membrane‐derived vesicles secreted by cells and released into the extracellular environment. EVs are mainly divided into three types: exosomes, microvesicles, and apoptotic bodies.^30^ Generally speaking, cells can secrete EVs through budding or fuse with the cytoplasmic membrane through endosomes, thus forming exosomes.^31^ Given the significant impact of exosomal circRNAs on tumor progression, the EVs mentioned in this article specifically refer to exosomes. EVs are composed of phospholipid bilayers and can be released into blood and urine.^32^ Currently, EVs are usually extracted from blood and the culture supernatants from cells.^33^ The ultracentrifugation method is the most commonly used way for extracting EVs, which can remove cell debris through low‐speed centrifugation and extract EVs through high‐speed centrifugation.^34^ The conventional circRNA detection methods are also applicable to circRNA in EVs, including PCR, microarray, high‐throughput sequencing, and northern blotting.^35^, ^36^, ^37^ Although researchers have studied various new methods for detecting exosomal miRNAs, further technological progress is still needed for the detection of circRNAs in EVs.^38^

Initially, EVs were seen as garbage bins for clearing redundant components within cells.^39^ With the increasing research on EVs, researchers have elucidated the crucial role of EVs in intercellular communication and their regulation of intracellular biological processes in various diseases.^40^, ^41^, ^42^ Exosomes start from endocytosis of the cell membrane and can produce early endosomes through the formation of buds within the cell. Subsequently, the early endosomes gradually mature and encapsulate proteins and nucleic acids, forming several intrinsic vessels and developing into multivesicular bodies.^43^ Finally, cell membrane fusion is mediated under the action of CD63, LAMP1, and LAMP2, followed by the release of EVs into the microenvironment.^44^

The contents of EVs mainly include DNA, mRNA, proteins, and ncRNA, which can act on receptor cells and regulate cancer progression.^45^, ^46^, ^47^ The content of these contents in exosomes may be closely related to the characteristics and progression of the disease.^48^ Previous studies on cancer have reported that EVs secreted by different tumor cells affect biological processes such as tumor growth, invasion, and drug resistance by fusing with receptor cell membranes.^49^, ^50^, ^51^ In addition, EVs have the ability to transmit antigens, regulate antigen presentation, and control tumor immune niches due to their surface facial membrane and special lumen structure. pMHCs (APCs present antigenic peptides) on EVs directly bind to antigen‐specific T cells, which can induce T cell activation, including T cell proliferation, cytotoxicity, and cytokine production.^52^, ^53^ In vivo studies have shown that EVs can also transmit EVs to DC cells, leading to the production of CD4^+^ and CD8^+^ T cells. Moreover, EVs derived from DC cells can stimulate T cell activity in the absence of antigen‐presenting cells (APCs) by carrying costimulatory molecules and pMHC.^54^, ^55^

Circular RNAs (circRNAs) are usually nonprotein coding RNAs that are spliced backward from their precursor mRNA into a loop.^56^ Due to its unique structure, circRNA can evade the action of exonucleases and has strong conservation, making it a potential biomarker for clinical applications.^57^, ^58^, ^59^ In the initial stage of tumors, the latest research has found that circRNAs can drive genetic mutations in hematological cancers, such as acute leukemia.^60^ CircRNAs can be enriched in MLL (the mixed linear leukemia) recombinants and bind to DNA, forming circRNA: DNA hybrids (circR loops). These circR loops promote chromosome recombination, induce mutations, and reduce genomic stability, which leads to the occurrence of acute leukemia.^61^ In addition, circRNAs are also involved in the stages of tumor metastasis. CircRNAs regulate the invasion, migration, angiogenesis, EMT process, and metastatic niche formation to achieve distant extravasation and colonization of cancer cells.^62^ The regulatory process of circRNAs can be achieved through various molecular mechanisms. Studies have demonstrated that circRNAs regulate tumor development through various molecular mechanisms.^63^ Most circRNAs can regulate downstream target proteins and pathways by competitively binding to miRNAs (also called competing endogenous RNA, ceRNA), thereby affecting the malignant progression of tumors.^64^ Certain circRNAs can directly interact with proteins to influence tumor growth, metastasis, and drug resistance.^65^ A small portion of circRNAs can form short peptides through translation and regulate cancer progression.^66^ Elucidating the molecular mechanisms of circRNA is crucial due to its involvement in biological processes like tumor growth and metastasis.

The biological process of circRNA encapsulation into EVs involves a large number of proteins, but its specific molecular mechanism remains unclear. It is speculated that the packaging of circRNAs into exosomes may be regulated by the following types of proteins: exosome‐related proteins, RNA binding proteins (RBPs), and transmembrane transporters.^67^, ^68^ Among them, exosome‐related proteins directly affect the synthesis of exosomal circRNAs by regulating membrane formation, circRNA loading and releasing.^69^ And RBPs can stabilize the generation of circRNA and regulate its entry into exosomes.^70^ Transmembrane transporters on EV membranes may regulate circRNA transport.^71^ Recent studies indicate that the protein hnRNPA2B1 can encapsulate various genetic materials (such as circRNAs) into exosomes.^72^, ^73^ For example, hnRNPA2B1 can directly bind to circNEIL3 and maintain the expression of circNEIL in exosomes, thereby delivering it to macrophages and promoting glioma development.^74^ In‐depth research on the biosynthesis, composition and function of exosomal circRNAs can further understand cancer progression and provide new targets and strategies for the diagnosis and treatment of tumors.

## 3 DOWNSTREAM SIGNALING PATHWAYS IN TUMORIGENESIS REGULATED BY circRNAs

CircRNAs alter the activity of various downstream signaling pathways, including JAK/signal transduction and transcription activation factor 3 (STAT3), MEK–ERK, PI3K/AKT, Wnt/β‐catenin, and Notch signaling pathways through these regulatory mechanisms in tumorigenesis.

### 3.1 JAK/STAT3 signaling pathway

**FIGURE 1:**
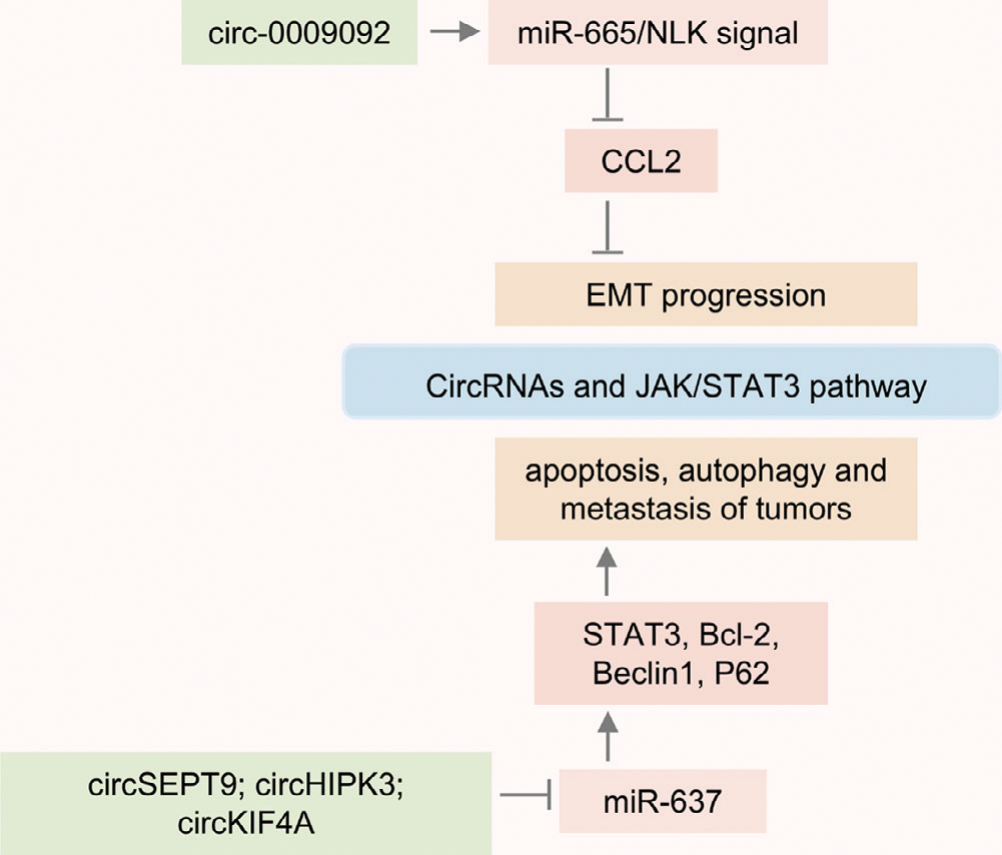
JAK/STAT3 signaling pathway regulated by circRNAs. Circ‐0009092 inhibits tumor progression by regulating the JAK/STAT3 pathway, while circSEPT9, circHIPK3, and circKIF4A activate STAT3 and promote cancer progression.

STAT3 plays a crucial role in cell proliferation, apoptosis, survival, and metastasis, acting as an oncogenic transcription factor.^75^ And STAT3 can act as a downstream target for various growth factors (TGF‐α/β, EGF, and PDGF) and cytokines (IL‐5, IL‐6, IL‐10, IL‐11, IL‐12, IL‐20, TNF‐α, and IFNγ).^76^ In recent years, research has found that circRNAs can regulate multiple target proteins of the STAT3 signaling pathway, and these STAT3‐related proteins are upregulated in various types of tumors.^77^, ^78^, ^79^ Circ‐0009092 regulates STAT3 modification and reduces CCL2 expression by targeting the miR‐665/NLK signaling axis, which inhibits tumor‐associated macrophage aggregation and slows down the EMT progression of tumor.^80^ In triple‐negative breast cancer (TNBC), circSEPT9 is synthesized with the participation of E2F1 and EIF4A3 proteins and is significantly upregulated in tumor tissues. Research indicates that inhibiting circSEPT9 can induce tumor cell apoptosis and inhibit tumor growth by downregulating the LIF/STAT3 signaling pathway.^81^ CircRNAs also affect autophagy by regulating the STAT3 signaling pathway. The expression of circKIF4A in brain metastasis from TNBC was significantly increased, and it could promote the expression of STAT3 and p62 through competitive adsorption of miR‐637, which induced tumor autophagy and metastasis.^82^ The expression of circHIPK3 is increased in tissues of colorectal cancer patients and is closely related to tumor volume, degree of metastasis, and survival.^83^ Also, researchers have identified that circHIPK3 targets the miR‐637/STAT3 signaling axis to upregulate Bcl‐2 and Beclin1 expression, thereby inhibiting autophagy and inducing oxaliplatin resistance in colorectal cancer.^84^ Therefore, STAT3 regulated by circRNA is involved in tumor cell proliferation, metastasis, apoptosis, and chemoresistance. And targeted inhibition of STAT3 activation has the potential to become one of the better strategies for reversing tumors (Figure 1).

### 3.2 MEK–ERK signaling pathway

The MEK–ERK signaling pathway is among the most extensively studied signaling cascades.^85^, ^86^ In humans, the effectors of signaling cascade reactions are mainly divided into three categories: (1) receptor tyrosine kinases of the HER family, such as RTKs, EGFR, and HER2‐4; (2) A/B/CRAF (serine/threonine kinase of the RAF family); (3) MEK1/2 and ERK1/2 (dual specificity protein kinase family).^87^, ^88^, ^89^ The inactivation of the ERK signaling pathway can lead to the development of autoimmune and neurodegenerative diseases, while the activation of the MEK–ERK pathway promotes cancer metastasis and drug resistance.^90^ Numerous evidence suggested that over 40% of cancers were closely related to dysregulation of the MEK–ERK pathway.^91^

CircRNAs in tumors can regulate the MEK–ERK signaling pathway and affect cancer progression.^92^, ^93^ CircPDIA4 is induced by the protein Quaking (the RBP) and inhibits the dephosphorylation of ERK1/2 by interacting with it, maintaining the high activity of the MAPK signaling pathway in the cytoplasm. In the nucleus, circPDIA4 interacts with DHX9, enhancing circRNA biosynthesis and promoting GC growth and metastasis.^94^ And circUBE4B encodes a new procancer peptide (circUBE4B‐173aa) and promotes the expression of phosphorylated MAPK1 by directly binding to MAPK1, which stimulates downstream MAPK/ERK signaling axis and promotes tumor malignant progression in the esophageal squamous cell carcinoma (ESCC).^95^ In addition, circRNAs affect chemoresistance by regulating the MEK–ERK signaling pathway in tumorigenesis. In TNBC, the overexpression of circZCCHC2 can lead to the decrease of miR‐1200 expression and the increase of TPR expression, which induces the activation of RAS–RAF–MEK–ERK cascade signaling and inhibits the sensitivity of tumor cells to pirarubicin therapy.^96^ In pancreatic ductal adenocarcinoma (PDAC), circRPS29 can directly bind to miR‐770‐5p and increase TRIM29 levels in vivo and in vitro, subsequently activating the downstream MEK/ERK signaling axis and inducing the emergence of gemcitabine resistance.^97^ Moreover, circPCNXL2 is significantly upregulated in the tissues of patients with intrahepatic cholangiocarcinoma (ICC) and promotes the interaction of STEP and MEK1/2 by directly binding to the protein STEP, thereby upregulating the ERK/MAPK axis to accelerate tumor progression and reducing the antitumor effect of trametinib.^98^ Thus, in‐depth research on the mechanism of ERK signaling dysregulation can provide new treatment options for alleviating tumor drug resistance.

### 3.3 PI3K/AKT signaling pathway

The PI3K/AKT signaling pathway initiates with PI3K activation, leading to elevated levels of PIP3 and AKT, thereby promoting tumor cell proliferation and survival.^99^, ^100^ The abnormal activation of the PI3K/AKT pathway enhances tumor cell migration, invasion and chemotherapy resistance.^101^, ^102^ CircRNAs can interact with the PI3K/AKT pathway. Specifically, circRNAs can directly bind to PI3K/AKT pathway‐associated proteins and affect tumor growth by regulating phosphorylation processes.^103^, ^104^, ^105^ CircZNF215 expression is significantly elevated in postoperative metastatic ICC tumors and is closely associated with patient prognosis. Moreover, circZNF215 competitively binds to PRDX1, inhibiting the interaction between PRDX1 and PTEN, thereby reducing the activity of the PTEN/AKT signaling pathway and promoting tumor metastasis as well as ipatasertib resistance.^106^ Moreover, circRNAs can modulate the activity of the PI3K/AKT pathway by competitively binding to miRNA. CircRPPH1 synthesized under the action of ZNF460 protein can regulate the expression of ITGA5 by directly binding to miR‐326, and then induce the activation of PI3K/AKT signal axis and accelerate the progress of TNBC.^107^ In addition, the latest research has found that a few circRNAs can also encode peptides and regulate tumor development by interacting with proteins in the PI3K/AKT pathway. CircTRIM1 can be translated into a new protein TRIM1‐269aa due to its IRES element and an 810  nt open reading frame. Functionally, circTRIM1 encoded TRIM1‐269aa enhances chemoresistance in TNBC by activating the PI3K/AKT/mTOR signaling pathway both in vivo and in vitro.^108^ CircPDE5A encodes a novel protein (PDE5A‐500aa) that directly binds to PIK3IP1, thereby reducing PI3K/AKT signaling activity and inhibiting tumor proliferation and metastasis in ESCC. In addition, researchers used the Meo–PEG–S–S–PLGA platform to load circPDE5A and protein PDE5A‐500aa for tumor therapy, and the results showed that it can effectively alleviate tumor progression.^109^ In general, further research on the circRNAs/PI3K/AKT signaling axis can provide new strategies for drug therapy and combination therapy in tumors.

### 3.4 Wnt/β‐catenin signaling pathway

The Wnt signaling pathway regulates diverse biological processes, including cell proliferation, cell death, cell cycle, stemness maintenance, and embryonic development.^110^, ^111^ The Wnt signaling pathway mainly includes two signal transduction pathways: classical and nonclassical pathways. Mutations in genes related to the Wnt signaling pathway, such as APC, RNF43, AXIN, and CTTB1, typically induce the development of multiple tumors. Also, mutations in FZD7, WNT11, WNT5A, VANGL1, and VANGL2 facilitate tumor cell migration, exacerbating cancers such as colorectal cancer, breast cancer, PCa, and ovarian cancer.^112^, ^113^, ^114^ In bladder cancer, circNIPBL is significantly elevated and enhances Wnt5a expression and Wnt/β‐catenin activity by directly binding to miR‐16‐2‐3p. The key protein ZEB1 in the Wnt/β‐catenin signaling pathway can bind to NIPBL pre‐mRNA, thereby relying on a positive feedback mechanism to promote the biosynthesis of circNIPBL and tumor growth.^115^ Overexpression of circFBXO7 in ovarian cancer cells can increase the level of MTSS1 by downregulating the level of miR‐96‐5p, inducing the accumulation and nuclear transport of β‐catenin, as well as phosphorylation of GSK3β, ultimately leading to the deterioration of ovarian cancer.^116^ Another circMMD enhances DVL1 levels by disrupting FIR and FUBP1 interaction and activates FZD6 expression by targeting miR‐15b‐5p. This results in continuous activation of the Wnt/β‐catenin signaling pathway, thereby worsening glioma malignancy.^117^ In addition, circRNAs target the Wnt/β‐catenin axis through binding with protein and then regulate the tumor progression. CircMTCL1 activates the expression of C1QBP and reduces the phosphorylation of β‐catenin protein by directly recruiting protein C1QBP, which activates the Wnt/β‐catenin signaling axis and exacerbates tumor growth and migration in laryngeal cancer.^118^ Reducing circ‐CCT2 expression diminishes TAF15 recruitment, destabilizes PTBP1 mRNA, and downregulates Wnt/β‐catenin axis activity, thereby influencing hepatoblastoma development.^119^ Thus, circRNAs lead to the abnormal activation of Wnt signaling, which promotes the malignant progression of various tumors.

### 3.5 Notch signaling pathway

The Notch signaling pathway exhibits high conservation during the evolutionary process, regulating tissue homeostasis, organ development, and determining cell fate.^120^ Recent scientific research indicates that the Notch signaling pathway can act as both a tumor suppressor and a protumor factor in cancer progression.^121^, ^122^ The dysregulation of the Notch signaling pathway enhances tumor invasiveness by accelerating angiogenesis and EMT progression.^123^, ^124^ And circRNAs participate in the regulation of the Notch signaling axis in tumor occurrence and development. Researchers conducted differential analysis between tumors and adjacent tissues and identified 11 circRNAs that were significantly differentially expressed in liver cancer. Among them, the expression of hsa_circ_001726 significantly increased in liver cancer tissues and cells, upregulating PRMT9 levels by binding to miR‐671‐5p. It subsequently enhanced liver cancer proliferation, metastasis, and EMT by activating the Notch signaling pathway.^125^ In addition, the upregulation of circ_0008532 in bladder cancer can inhibit the expression of miR‐330‐5p and miR‐155‐5p, induce the level of downstream target gene MTGR1 and activate Notch signal axis, which enhances the invasive ability of tumors.^126^ Moreover, the absence of circFBXW7 promotes leukemia cell proliferation by regulating MYC and NOTCH1 protein levels in both in vivo and in vitro experiments, indicating its crucial role in the progression of T‐cell acute lymphoblastic leukemia.^127^ A few circRNAs inhibit tumor growth and metastasis by regulating the Notch signaling pathway. Recent studies indicate that circCRIM1 absorbs miR‐146a‐5p, leading to the upregulation of NUMB, which inhibits Notch signaling and reduces osteosarcoma (OS) cell proliferation, invasion, and migration.^128^ Therefore, circRNAs regulate the Notch signaling pathway in the TME, balancing protumor and tumor‐suppressive effects, and offering new therapeutic strategies for malignant tumors (Table 1).

**TABLE 1:** Downstream signaling pathways regulated by circRNAs in tumorigenesis.

Pathways	CircRNAs	Cancer type	Expression	Targets	References
JAK/STAT3	circ‐0009092	Colorectal cancer	Down	miR‐665/NLK	80
	circSEPT9	TNBC	Up	miR‐637/LIF/STAT3	81
	circKIF4A	TNBC	Up	miR‐637/p62	82
	circHIPK3	Colorectal cancer	Up	miR‐637/STAT3	84
MEK–ERK	circPDIA4	Gastric cancer	Up	ERK1/2/STAT3	94
	circUBE4B	ESCC	Up	circUBE4B‐173aa	95
	circZCCHC2	TNBC	Up	miR‐1200/TPR	96
	circRPS29	PDAC	Up	miR‐770‐5p/TRIM29	97
	circPCNXL2	ICC	Up	STEP/MEK1/2	98
PI3K/AKT	circZNF215	ICC	Up	PRDX1/PTEN	106
	circRPPH1	TNBC	Up	miR‐326/ITGA5	107
	circTRIM1	TNBC	Up	TRIM1‐269aa	108
	circPDE5A	ESCC	Down	PDE5A‐500aa	109
Wnt/β‐catenin	circNIPBL	Bladder cancer	Up	miR‐16‐2‐3p/Wnt5a	115
	circFBXO7	Ovarian cancer	Up	miR‐96‐5p/MTSS1	116
	circMMD	Glioma	Up	miR‐15b‐5p/FZD6	117
	circMTCL1	Laryngeal cancer	Up	C1QBP/β‐catenin	118
	circ‐CCT2	Hepatoblastoma	Up	TAF15/PTBP1	119
Notch	hsa_circ_001726	Liver cancer	Up	miR‐671‐5p/PRMT9	125
	circ_0008532	Bladder cancer	Up	miR‐330‐5p/miR‐155‐5p	126
	circFBXW7	T‐cell acute lymphoblastic leukemia	Down	MYC/NOTCH1	127
	circCRIM1	Osteosarcoma	Down	miR‐146a‐5p/NUMB	128

## 4 CHARACTERISTICS AND FUNCTIONS OF EXOSOMAL circRNAs

**FIGURE 2:**
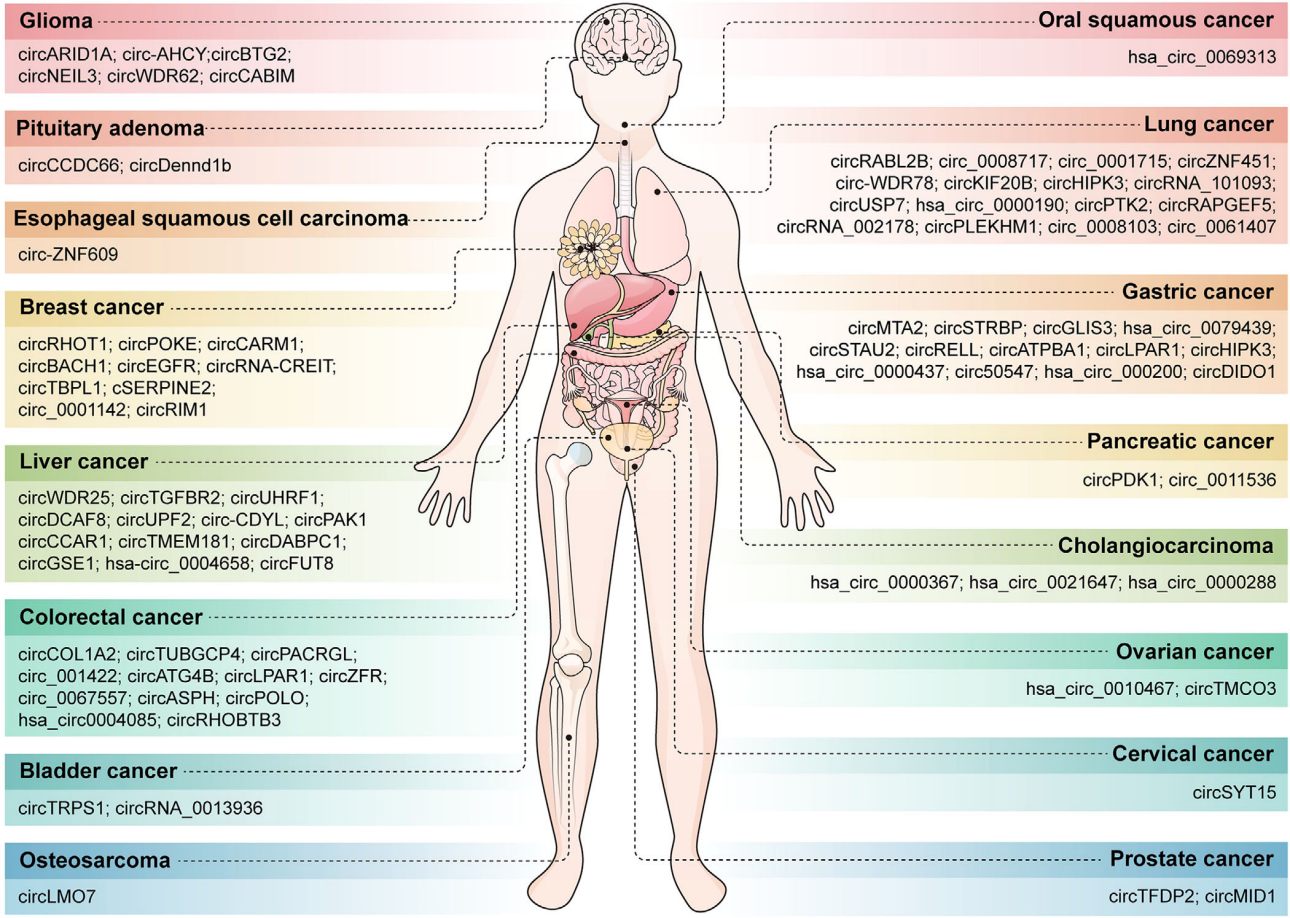
Exosomal circRNAs in different types of cancer. The exosomal circRNAs are differentially expressed in many types of tumors, including glioma, pituitary adenoma, esophageal squamous cell carcinoma, breast cancer, liver cancer, colorectal cancer, bladder cancer, osteosarcoma, oral squamous cancer, lung cancer, gastric cancer, pancreatic cancer, cholangiocarcinoma, ovarian cancer, cervical cancer, and prostate cancer.

Exosomes carry various genetic molecules, including circRNAs. Numerous studies have demonstrated that the expression of circRNAs in exosomes is significantly dysregulated during tumor progression, which can affect tumor occurrence and development by regulating biological processes such as proliferation, metastasis, EMT progression, autophagy, glycolysis, and chemotherapy resistance (Figure 2).^129^, ^130^

### 4.1 Exosomal circRNAs regulate tumor proliferation and metastasis

Exosomal circRNA plays an oncogenic role in different types of tumors, which promotes tumor growth and metastasis. CircMTA2 is a circRNA transmitted by exosomes that can inhibit the ubiquitination process of MTA2 protein and stabilize the MTA2 structure by directly binding to UCHL3, thereby exacerbating the occurrence and invasion of GC.^131^ In addition, circ_0008717 in EVs can activate the levels of downstream target PAK2 in a miR‐1287‐5p‐dependent manner, promoting the occurrence and metastasis of NSCLC.^132^ And the exploration in an in vivo xenograft tumor model indicates that the exosomal circSTRBP derived from GC cells was found to activate downstream E2F2 (E2F transcription factor 2) and enhance the progression of GC by absorbing miR‐593‐3p and miR‐1294.^133^ Moreover, researchers screened circARID1A with significantly increased expression in glioblastoma through array analysis and found that circARID1A is abundant in exosomes. In‐depth exploration revealed that exosomal circARID1A accelerates tumor invasion and migration by absorbing miR‐370‐3p and upregulating TGFBR2 levels.^134^

Certain exosomal circRNAs can suppress tumor cell proliferation and migration. In OS, the exosomal circLMO7 relies on miR‐21‐5p to increase the level of ARHGAP24, which acts as a tumor suppressor to slow down cancer progression.^135^ CircSTAU2, transmitted via exosomes, regulates CAPZA1 expression by absorbing miR‐589, thereby inhibiting GC progression.^136^ Also, circPOKE exists in the exosomes of breast cancer cells and can reduce its binding with snail (the crucial regulator of the EMT process) by directly binding to USP10 protein, thus inhibiting the metastasis of breast cancer cells rather than proliferation.^137^

### 4.2 Exosomal circRNAs regulate angiogenesis and EMT progression

Exosomal circRNAs can regulate tumor migration and invasion through their regulatory role in angiogenesis. CircTUBGCP4 derived from exosomes of colorectal cancer cells can increase PDK2 expression and promote AKT signaling pathway activation by targeting miR‐146b‐3p, thereby promoting endothelial cell migration and angiogenesis.^138^ And the exosomal circ‐WDR78 mediates the inactivation of the HIF1‐α signaling pathway and inhibits the progression of NSCLC cells by absorbing miR‐1265 and regulating the expression of FBXW8.^139^ Also, the expression of circ_001422 in serum exosomes of colorectal cancer patients is significantly increased and positively correlated with the degree of lymph node metastasis. And the exosomal circ_001422 increases the proliferation and migration process of endothelial cells and activates KDR/mTOR signaling and colorectal cancer progression by inhibiting miR‐195‐5p activity.^140^ Under hypoxic conditions, circ‐ZNF609 in EVs of ESCC elevates VEGFA (vascular endothelial growth factor A) by decreasing miR‐150‐5p levels and directly binds to HuR protein to inhibit the translation of ZO‐1, Occludin, and Claudin‐1 mRNA, thereby exacerbating cancer progression.^141^

Also, the exosomal circRNAs transmitted between tumor cells can activate the metastasis of tumors by regulating the EMT progression. CircCOL1A2 is overexpressed in colorectal cancer cells and can be encapsulated in exosomes, facilitating tumor cell proliferation, metastasis, and EMT progression. Further mechanistic studies have found that the exosomal circCOL1A2 increases the expression of LASP1 by binding to miR‐665, exacerbating the progression of colorectal cancer.^142^ Also, circRHOT1 in exosomes directly targets miR‐204‐5p, promoting PRMT5 expression and accelerating the growth, migration and EMT progression of breast cancer cells in both in vivo and in vitro experiments.^143^ Moreover, exosomal circWDR25 derived from hepatic stellate cells can upregulate the expression of ALOX15 in a miR‐4474‐3p‐dependent manner, thereby promoting EMT and exacerbating the invasive characteristics of hepatocellular carcinoma (HCC).^144^

### 4.3 Exosomal circRNAs regulate autophagy and glycolysis

Autophagy can also serve as one of the most important biological processes regulated by exosomal circRNAs, thereby affecting the malignant progression of tumors. Exosomal circRELL activates autophagy by regulating the miR‐637/EPHB3 signaling axis, thereby inhibiting the proliferation and metastasis of GC cells in both in vivo and in vitro experiments.^145^ Under the stress of starvation, the exosomal circTGFBR2 secreted by liver cells can competitively bind to miR‐205‐5p and increase the expression of ATG5, which induces protective autophagy in HCC cells and promotes tumor progression.^146^ During amino acid starvation‐induced autophagy, exosomal circEGFR activates autophagy by regulating ANXA2 membrane translocation and forms a negative feedback loop to modulate autophagy in TNBC by targeting the miR‐224‐5p/ATG13/ULK1 signaling pathway.^147^

In addition, recent studies indicate that a subset of exosomal circRNAs can regulate metabolic processes like glycolysis, thereby influencing tumor development. Exosomal circCARM1 secreted by breast cancer stem cells targets the miR‐1252‐5p/PFKFB2 signaling axis, reprogramming tumor cell glycolysis.^148^ Also, the expression of circPDK1 is increased in pancreatic cancer tissues and serum exosomes, and it is related to the poor prognosis of patients. And exosomal circPDK1, activated by HIF1A, promotes migration and glycolysis of pancreatic cancer by inducing the activity of miR‐628‐3p/BPTF signal and the degradation of BIN1 protein.^149^

### 4.4 Exosomal circRNAs regulate chemoresistance

**FIGURE 3:**
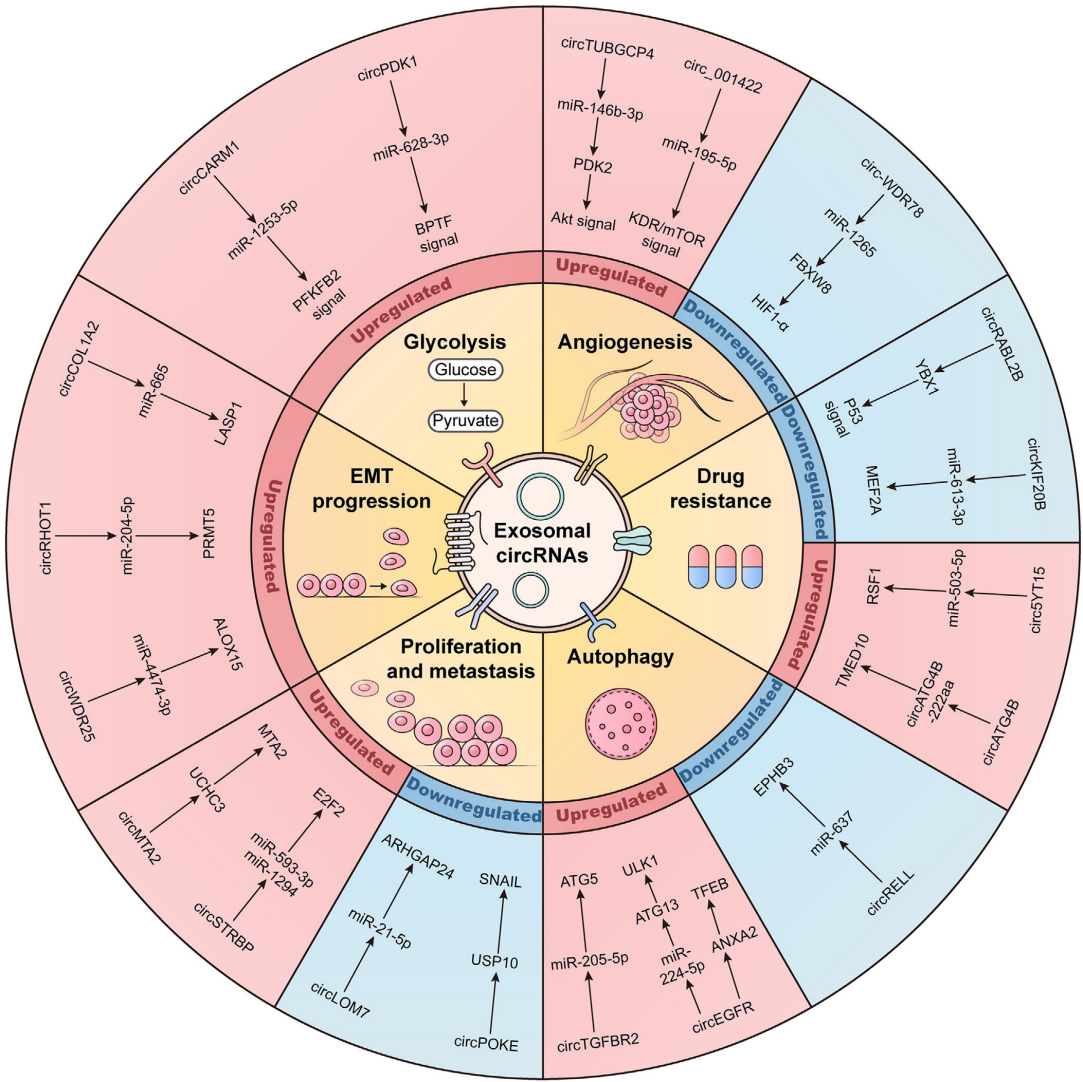
Function of exosomal circRNAs in tumorigenesis. Exosomal circRNAs can regulate the activity of downstream target genes and signaling pathways, subsequently affecting various tumor‐related biological processes, including proliferation, metastasis, EMT progression, angiogenesis, glycolysis, autophagy, and drug resistance.

One of the most important challenges in current cancer therapy is chemoresistance.^150^ More and more studies have elucidated the close correlation between exosomal circRNAs, chemoresistance, and malignant progression of tumors (Figure 3).^151^, ^152^ Oxaliplatin resistance significantly affects the treatment efficacy in colorectal cancer patients.^153^ Researchers have found that the exosomal circATG4B is upregulated in oxaliplatin‐resistant cells and regulates the expression of the TMED10 gene by encoding the circATG4B‐222aa protein, leading to increased autophagy and induction of drug resistance.^154^ The elevated hsa_circ_0010467 levels in drug‐resistant ovarian cancer tissues, cells, and serum exosomes can predict tumor grading and prognosis. Mechanistically, researchers have found that hsa_circ_0010467 maintains platinum resistance and promotes the growth of ovarian cancer by mediating downregulation of miR‐637 and upregulation of LIF/STAT3 axis.^155^ And circSYT15 in EVs can competitively bind to miR‐503‐5p, regulate RSF1 expression, and promote cell proliferation and cisplatin resistance in cervical cancer.^156^ Moreover, exosomal circDCAF8 enhances HCC cell growth and metastasis via the miR‐217/NAP1L1 signaling axis, promotes vascular endothelial cell angiogenesis, and induces regorafenib resistance.^157^ And circUPF2 in EVs can stabilize the expression of SLC7A11 by forming complexes with IGF2BP2 and SLC7A11, which reduces the sensitivity of HCC cells to ferroptosis and promotes resistance to sorafenib.^158^ Also, paclitaxel treatment significantly increased circBACH1 expression in exosomes of breast cancer cells. At the same time, exosomal circBACH1 enhances breast cancer cell stemness, angiogenesis, and metastasis by targeting the miR‐217/G3BP2 axis, thereby promoting paclitaxel resistance and advancing breast cancer progression.^159^

The increase of several exosomal circRNAs can also enhance the sensitivity of chemotherapy in tumor cells. Researchers have found that overexpression of circRNA–CREIT can weaken resistance to doxorubicin treatment by degrading PKR proteins and promoting the activity of the apoptosis‐related signaling pathway RACK1/MTK1. CircRNA–CREIT in exosomes can be transferred to tumor cells, potentially serving as an effective strategy to mitigate chemoresistance in TNBC.^160^ In patients resistant to gefitinib, the expression of circKIF20B in serum exosomes is significantly reduced and negatively correlated with tumor stage. Functionally, exosomal circKIF20B binds to miR‐615‐3p, modulating MEF2A expression, which inhibits the cell cycle, promotes apoptosis, reduces mitochondrial oxidative phosphorylation, and increases gefitinib sensitivity in NSCLC.^161^ Also, exosomal circRABL2B binds to YBX1, downregulates MUC5AC expression, reduces integrin β4/pSrc/p53 signaling pathway activity, inhibits tumor cell stemness, and alleviates erlotinib resistance in lung cancer.^162^

In short, exosomal circRNAs are transmitted between various cells and affect tumor malignant progression by regulating cancer‐related biological processes and signaling pathways (Table 2), which provides potential molecular targets for tumor diagnosis and treatment.

**TABLE 2:** Function of exosomal circRNAs in various cancer types.

Exosomal circRNAs	Cancer type	Expression	Target	Function	References
circMTA2	Gastric cancer	Up	MTA2	Promote the proliferation and metastasis	131
circ_0008717	NSCLC	Up	miR‐1287‐5p	Promote the occurrence and metastasis	132
circSTRBP	Gastric cancer	Up	miR‐593‐3p/ miR‐1294	Enhance GC cell growth and migration	133
circARID1A	Glioblastoma	Up	miR‐370‐3p	Accelerate tumor invasion and migration	134
circLMO7	OS	Down	miR‐21‐5p	Suppress the growth and migration	135
circSTAU2	Gastric cancer	Down	miR‐589	Inhibit proliferation and migration	136
circPOKE	Breast cancer	Down	snail	Inhibit the metastasis of breast cancer cells rather than proliferation	137
circTUBGCP4	Colorectal cancer	Up	miR‐146b‐3p	Promote endothelial cell migration	138
circ‐WDR78	NSCLC	Down	miR‐1265	Induce ROS accumulation; inhibit NSCLC cell proliferation, invasion, and migration	139
circ_001422	Colorectal cancer	Up	miR‐195‐5p	Promote endothelial cell migration	140
circ‐ZNF609	ESCC	Up	miR‐150‐5p	Facilitate angiogenesis and vascular permeability; enhance distant metastasis	141
circCOL1A2	Colorectal cancer	Up	miR‐665	Promote proliferation and EMT progression	142
circRHOT1	Breast cancer	Up	miR‐204‐5p	Accelerate growth and EMT progression	143
circWDR25	HCC	Up	miR‐4474‐3p	Promote EMT and invasive characteristics	144
circRELL	Gastric cancer	Down	miR‐637	Activates autophagy; inhibit proliferation	145
circTGFBR2	HCC	Up	miR‐205‐5p	Promote HCC progression	146
circEGFR	TNBC	Up	miR‐224‐5p/ ANXA2	Promote autophagy, malignant progression	147
circCARM1	Breast cancer	Up	miR‐1252‐5p	Reprogram the glycolysis process	148
circPDK1	Pancreatic cancer	Up	miR‐628‐3p	Promote migration and glycolysis	149
circATG4B	Colorectal cancer	Up	circATG4B‐222aa	Increase autophagy and induce oxaliplatin resistance	154
hsa_circ_0010467	Ovarian cancer	Up	miR‐637	Maintain platinum resistance and promote ovarian cancer growth	155
circSYT15	Cervical cancer	Up	miR‐503‐5p	Promote cell proliferation and cisplatin resistance	156
circDCAF8	HCC	Up	miR‐217	Promote angiogenesis and regorafenib resistance	157
circUPF2	HCC	Up	IGF2BP2	Reduce ferroptosis sensitivity and promote sorafenib resistance	158
circBACH1	Breast cancer	Up	miR‐217	Enhance the stemness, angiogenesis, and metastasis; promote paclitaxel resistance	159
circRNA–CREIT	TNBC	Down	PKR	Weaken resistance to doxorubicin; promote the activity of the apoptosis	160
circKIF20B	NSCLC	Down	miR‐615‐3p	Exacerbate gefitinib sensitivity	161
circRABL2B	Lung cancer	Down	YBX1	Inhibit stemness and erlotinib resistance	162
circ‐AHCY	Glioblastoma	Up	miR‐1294	Promote GBM cell growth	169
circ‐0011536	PDAC	Up	miR‐451a	Promote tumor growth and increase peripheral nerve invasion and remodeling	170
circTFDP2	Prostate cancer	Up	PARP1	Promote proliferation and metastasis	171

Abbreviations: GBM, glioblastoma; ROS, reactive oxygen species.

## 5 EXOSOMAL circRNAs IN TME

The TME is a complex structure comprising various cells, exosomes, and extracellular matrix.^163^ The cells in TME mainly include tumor cells, various types of immune cells, cancer‐related fibroblasts, pericytes, endothelial cells, and other tissue‐resident cells.^164^ Due to their synergistic function in the TME, these cells form ecological niches that promote or inhibit tumor progression, thereby regulating the growth, migration, and invasion processes of tumors.^165^ Therefore, an in‐depth discussion of the interactions between different types of cells in the microenvironment is of great significance for explaining tumor characteristics and finding therapeutic targets.

**FIGURE 4:**
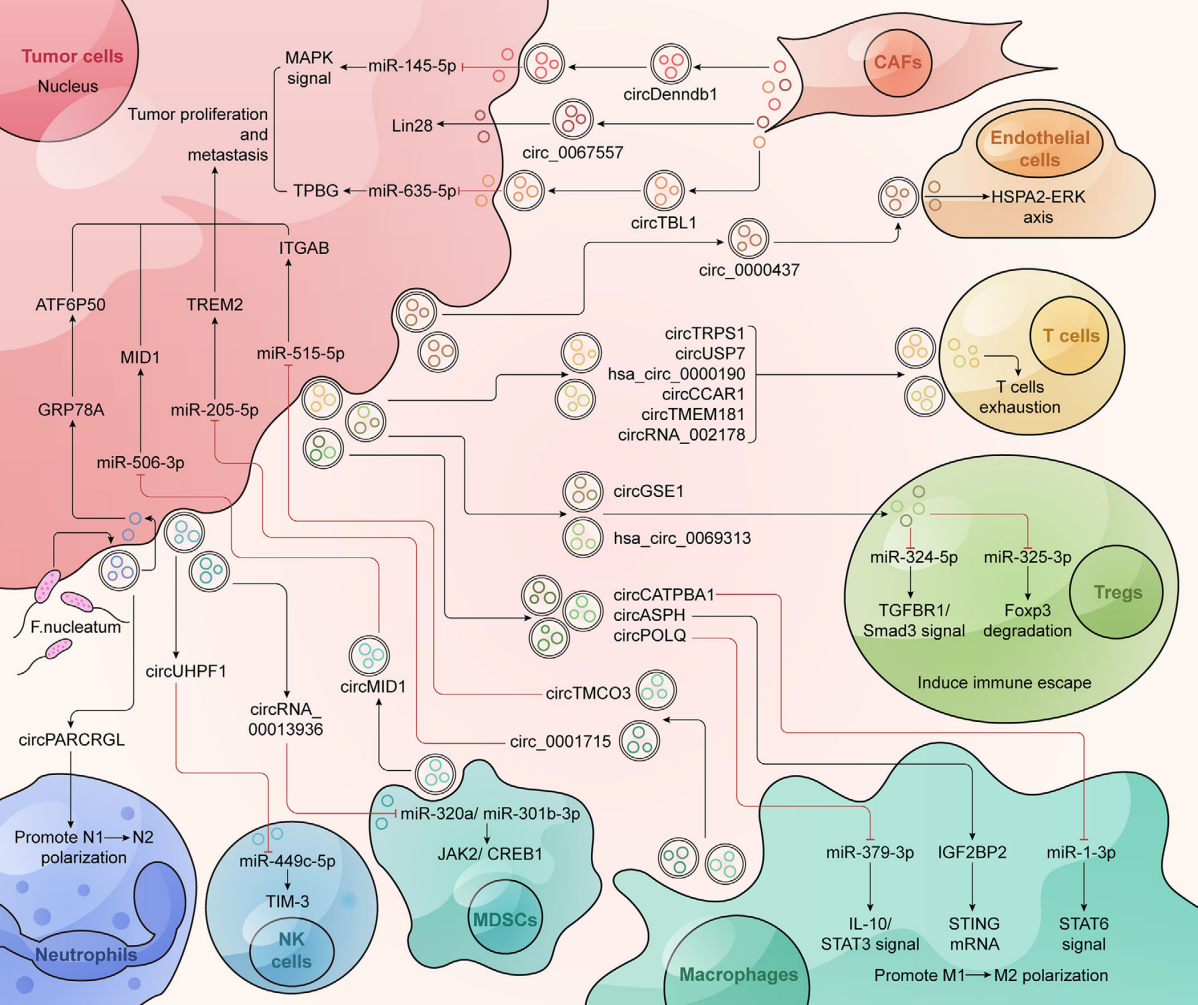
Exosomal circRNAs facilitate communication in various cells of the tumor microenvironment. The cell types in the tumor microenvironment mainly include tumor cells, CAFs, endothelial cells, T cells, Treg cells, macrophages, MDSCs, NK cells, and neutrophils. Multiple cells reshape the immune microenvironment and affect tumor progression by transmitting exosomal circRNAs. In addition, a few gut microbiota can influence the synthesis of exosomal circRNAs in tumor cells, thereby regulating tumor development.

Exosomes in the TME are produced by paracrine cell types and can transmit various genetic molecules, including circRNAs. Usually, donor cells encapsulate circRNAs into exosomes and transport them to recipient cells, thus participating in intercellular communication.^166^ Tumor cells can promote tumor progression by transferring exosomal circRNAs to immune cells or activating nearby fibroblasts. Immune cells such as macrophages, T cells, and NK cells can communicate with tumor cells by transmitting exosomal circRNA to regulate immune cell function and promote the formation of an immunosuppressive microenvironment. For example, exosomal circRNA, as an important regulatory molecule for immune escape, can reduce T cell activation and enhance the immune tolerance of tumor cells.^167^ In the TME, angiogenesis is crucial for tumor proliferation and migration, and exosomal circRNA plays an important role in angiogenesis. Exosomal circRNAs can reshape the TME and activate cancer progression by competitively binding to miRNA, interacting with proteins and affecting endothelial cell function. In addition, exosomal circRNAs in the TME can also induce tumor cells to develop chemoresistance by regulating resistance‐related molecular pathways.^168^ CircRNAs in exosomes facilitate information exchange between different types of cells in the TME, playing an important role in cancer metabolism and immune regulation (Figure 4).

### 5.1 Tumor‐derived exosomal circRNAs act on themselves

In the TME, tumor cells secrete exosomes that transmit genetic material (including circRNAs) to themselves or distant tumor cells, thus regulating tumor progression. Exosomal circRNAs regulate tumor‐related signaling pathways and biological processes to influence the malignancy of tumors. Exosomal circ‐AHCY can absorb miR‐1294, leading to increased MYC and CTNNB1 expression and subsequently activating the Wnt/β‐catenin signaling pathway. At the same time, it can also recruit EIF4A3 and stabilize TCF4 and β‐catenin protein, which increases the expression of circ‐AHCY through a positive feedback axis.^169^ In addition, Hedgehog‐Gli1 mediates an increase in the expression of circ‐0011536 in tumor exosomes, followed by targeting miR‐451a/VGF signaling to promote tumor growth and increase peripheral nerve invasion and remodeling in PDAC.^170^ And circRNAs in exosomes can also affect DNA damage in tumor cells. Exosome‐derived circTFDP2 is upregulated in PCa tissue and inhibits the cleavage of active caspase‐3 by interacting with PARP1 protein, slowing down DNA damage in PCa and promoting cancer progression.^171^

Also, exosomes secreted by higher metastatic tumor cells can induce an advanced phenotype in lower metastatic tumor cells. Chen et al.^172^ demonstrated that exosomes from highly metastatic HCC cells can transfer to lower metastatic tumor cells, activating MAPK/ERK signals and intensifying EMT, thereby enhancing the metastatic potential of the lower metastatic cells. Among them, the exosomes derived from HCC cells with higher metastatic potential are rich in circRNA‐100338. An in vitro experiment demonstrated that overexpression of exosomal circRNA‐100338 can significantly enhance the migration and invasion abilities of HCC cells.^173^ In a word, these studies have found that exosomal circRNAs secreted by tumor cells have been shown to act on themselves and participate in regulating tumor growth and metastasis.

### 5.2 Exosomal circRNAs and CAFs

CAFs, derived from activated fibroblasts, are crucial components of the TME.^174^ CAFs can be categorized into myCAFs (characterized by myofibroblast traits and elevated α‐SMA and FAP expression) and iCAFs (inflammatory CAFs, which secrete factors and regulate inflammation).^175^, ^176^ CAFs can interfere with tumor treatment and predict tumor prognosis by activating various cancer‐related signals. Meanwhile, the transmission of exosomes between CAFs and tumor cells contributes to the formation of the TME and exacerbates tumor progression.^177^ By high‐throughput sequencing of normal fibroblasts and CAFs in breast cancer, researchers screened the exosomal circTBPL1 that is significantly upregulated in CAFs. The exosomal circTBPL1 secreted by CAFs is absorbed by tumor cells and promotes TPBG expression by competitively binding to miR‐653‐5p, thereby accelerating breast cancer proliferation and invasion.^178^ Another study in pituitary adenomas found that circDennd1b in EVs derived from CAFs can inhibit miR‐145‐5p and upregulate ONECUT2 expression, subsequently activating the activity of the MAPK signaling pathway and promoting tumor metastasis.^179^ In colorectal cancer, exosomal‐transported circ_0067557 derived from CAFs can regulate Lin28 (including Lin28A and Lin28B), which enhances proliferation, metastasis, and chemoresistance of tumor cells.^180^ Thus, exosomal circRNAs derived from CAFs are transported to tumor cells and regulate tumor progression by targeting downstream signaling pathways within tumor cells.

### 5.3 Exosomal circRNAs and endothelial cells

The endothelial‐vesicular network is a novel mechanism regulating the tumor vascular system and microenvironment by altering the Notch signaling pathway and VEGFR2/3.^181^, ^182^ VEGFR3 is one of the key factors in lymphangiogenesis, which can increase the activity of lymphatic endothelial cells and promote their migration.^183^ Endothelial cells establish intercellular communication by releasing and absorbing EVs. Researchers have found that EVs secreted by endothelial cells can deliver protein DLL4 to nearby endothelial cells and improve their migration ability by regulating the Notch signaling pathway.^184^ Moreover, the EVs of endothelial cells can also load miRNAs and transfer them into tumor cells, affecting the sensitivity of chemotherapy.^185^, ^186^ In addition, the exosomal circRNAs derived from tumor cells can be taken up by endothelial cells, enhancing tumor metastasis by promoting endothelial cell migration. Exosomes secreted by GC cells transport hsa_circ_0000437 to human lymphatic endothelial cells. It promotes the formation of lymphatic vessels and lymph node metastasis by targeting the HSPA2–ERK cascade signal, which may become a new biomarker for predicting the prognosis and targeted treatment of GC.^187^ In a word, the vesicular transport of endothelial cells affects tumor angiogenesis and metastasis, which can become a promising new strategy for tumor therapy.

### 5.4 Exosomal circRNAs and T cells

The dysfunction of CD8^+^ T cells characterizes the tumor immune microenvironment.^188^, ^189^ This inhibitory tumor immune microenvironment is accompanied by depletion of CD8^+^ T cells, reduced cytokine release, and decreased cytotoxic function.^190^, ^191^, ^192^ Exosomal circRNAs regulate drug resistance in tumor immunotherapy across various cancer types. In lung cancer, circRNA‐002178 is present in plasma exosomes of lung adenocarcinoma (LUAD) patients and upregulates PD‐L1 levels by competitively binding to miR‐34. Meanwhile, circRNA‐002178, which relies on EVs for transmission, can be absorbed by CD8^+^ T cells and induce an increase in PD1, ultimately depleting T cells and promoting lung cancer development.^193^ And exosomal circUSP7 derived from NSCLC cells targets the miR‐934/SHP2 axis, causing CD8^+^ T cell dysfunction and increasing resistance to anti‐PD1 therapy in NSCLC patients.^194^ Another hsa_circ_0000190 is present in tissues, blood, urine, and exosomes of NSCLC patients. It can upregulate the level of sPD‐L1 (soluble PD‐L1), reduce the function of PD‐L1 antibodies, and inhibit T cell activation, which promotes the emergence of immune therapy drug resistance and leads to poor prognosis in patients.^195^ In HCC cells, researchers have confirmed that circCCAR1 increases the expression of WTAP by absorbing miR‐127‐5p and is packaged into exosomes with the involvement of hnRNPA2B1 through the use of chromatin immunoprecipitation, RNA immunoprecipitation, and biotinylated RNA pull‐down assays. Then, researchers explored the role of exosomal circCCAR1 in anti‐PD1 resistance by constructing a mouse model containing recombinant human immune system components. It was found that circCCAR1 in EVs stabilizes PD‐1 protein in CD8^+^ T cells, causing their dysfunction and enhancing resistance to anti‐PD1.^196^ Also, researchers have found that circTMEM181 is significantly upregulated in anti‐PD1‐resistant HCC patients. Exosomal circTMEM181 enhances CD39 expression in macrophages by targeting miR‐488‐3p. The coexpression of CD39 in macrophages and CD73 in HCC cells enhances adenosine production, which inhibits CD8^+^ T cell activity and induces anti‐PD1 resistance.^197^ In bladder cancer, the exosomal circTRPS1 directly combines with miR‐141‐3p to regulate GLS1‐dependent glutamine metabolism, balance the level of ROS, and deplete CD8^+^ T cells in the TME, thus promoting the malignant progression of tumors.^198^

In addition, regulatory T cells (Tregs) produce immunosuppressive molecules, inhibiting CD8^+^ T cell activity and facilitating tumor immune escape.^199^, ^200^, ^201^ Exosomal circGSE1 regulates the TGFBR1/Smad3 signaling axis by targeting miR‐324‐5p, subsequently increasing the number of Tregs and promoting the progression of HCC.^202^ Has_circ_0069313 is upregulated in oral squamous cancer cells and induces immune escape by leading to the reduction of miR‐325‐3p expression and activating the degradation of Foxp3. Also, has_circ_0069313 in exosomes is transferred to Tregs and maintains the function of Treg cells.^203^ Altogether, exosomal circRNAs affect the efficacy of immunotherapy and tumor progression by regulating the function of T cells and Treg cells.

### 5.5 Exosomal circRNAs and macrophages

Macrophages are essential for antigen presentation and initiating inflammatory responses, positioning them as a key immune population in the body.^204^ Macrophages are usually divided into two subtypes: M1 and M2. Among them, tumor‐associated macrophages have an M2 phenotype and account for about half of the tumor mass.^205^ Previous studies have identified that tumor‐associated M2 macrophages and their secreted exosomes significantly contribute to tumor progression.^206^, ^207^ In ovarian cancer, the exosomal circTMCO3 is derived from M2 macrophages that competitively bind to miR‐515‐5p and increase the expression of ITGA8, thereby promoting malignant progression.^208^ Several exosomes derived from M1 macrophages contribute to antitumor therapy by modulating circRNA expression. Exosomes from M1 macrophages deliver miR‐628‐5p to liver cancer cells, inhibiting METTL14 expression and enhancing circFUT8 transport from the nucleus to the cytoplasm. Subsequently, circFUT8 inhibits the occurrence and development of liver cancer by targeting the miR‐552‐3p/CHMP4B signaling axis.^209^ In addition, overexpression of circ_0001715 in M0 macrophages can be transmitted to LUAD cells through exosomes and can also induce macrophage polarization toward M2 type and exacerbate lung cancer metastasis by targeting miR‐205‐5p and increasing TREM2 levels.^210^ Moreover, the expression of hsa_circ_0004658 in exosomes from RBPJ‐activated macrophages was significantly elevated, and this exosomal hsa_circ_0004658 inhibited liver cancer progression by modulating miR‐499b‐5p and JAM3 expression.^211^ Also, the exosomal circBTG2 derived from activated macrophages can regulate the expression of PTEN protein by directly binding to miR‐25‐3p, and subsequently inhibit the growth and invasion of glioma.^212^

A large amount of exosomal circRNAs from tumor cells can modulate macrophage transition from M1 to M2 types and influence tumor malignancy. Recent studies indicate that the exosomal circATP8A1 is significantly overexpressed in the plasma of GC patients and is closely associated with TNM stage and prognosis. CircATP8A1 in EVs can activate the STAT6 signaling pathway rather than the STAT3 signaling pathway by targeting miR‐1‐3p, thereby activating the appearance of M2 macrophages and inducing cancer progression.^213^ And circNEIL3 relies on the action of hnRNPA2B1 protein to package into exosomes and stabilize the expression of IGF2BP3 protein through delivery to tumor‐associated macrophages, producing immunosuppressive effects and subsequently promoting the growth of gliomas.^74^ Another exosomal circASPH enhances the stability of STING mRNA by directly binding to IGF2BP2, thereby increasing the transition of macrophages from M1 to M2 and promoting tumor growth.^214^ The exosomal cSERPINE2 from tumor cells acts on the tumor‐associated macrophages and promotes the secretion of IL‐6, thereby accelerating the proliferation and metastasis of breast cancer cells. At the same time, enhances EIF4A3 and CCL2 expression via positive feedback, induces cSERPINE2 production, and further recruits tumor‐associated macrophages in breast cancer.^215^ And the exosomal circPOLQ derived from tumor cells increases IL‐10 and STAT3 by absorbing miR‐379‐3p, thereby promoting polarization of M2 macrophages and exacerbating the occurrence of metastatic nodules in colorectal cancer.^216^ Also, circGLIS3 in tumor cells regulates the miR‐1343‐3p/PGK1 axis and directly binds to VIMENTIN, promoting GC proliferation and invasion both in vivo and in vitro. Additionally, circGLIS3 can be encapsulated into EVs and transmitted to macrophages, which induces polarization of macrophages toward M2 type.^217^

The exosomal circRNAs induced by stress also participate in the polarization of macrophages from M1 to M2. Recent research indicates that endoplasmic reticulum (ER) stress enhances tumor cell exosome secretion, facilitates the transfer of circ_0001142 into macrophages, and modulates the miR‐361‐3p/PIK3CB pathway, thereby affecting macrophage autophagy and polarization in breast cancer.^218^ And, exosomal circPLEKHM1 is produced by tumor cells under hypoxic conditions and can interact with macrophages in the TME. Specifically, circPLEKHM1 exacerbates tumor metastasis by enhancing the interaction between PAPC1 and eIF4G, increasing the translation of OSMR, and driving macrophage polarization toward the M2 type in NSCLC.^219^ Moreover, Kras mutation induces heterogeneity within tumors by increasing the expression of exosomal circHIPK3 and circPTK2, which induces the appearance of M2 macrophages, immune suppression, and exacerbates lymph node metastasis in lung cancer.^220^ Therefore, exosomal circRNAs derived from macrophages act on tumor cells and regulate tumor malignant progression, while the exosomal circRNAs secreted by tumor cells can be absorbed by macrophages and induce the appearance of M2 macrophages.

### 5.6 Exosomal circRNAs and NKs

NK cells similar to CD8^+^ T cells have the ability to kill target cells and are important effector cells in innate immunity.^221^ NK cells constitute approximately 5–10% of PBMCs (whole blood monocytes) and are primarily found in the blood, spleen, and bone marrow. The growth of NK cells typically requires transcription factors (including Tox, Nfil3, Id2, T‐bet, and EOMES) and cytokines (including IL‐2 and IL‐15).^222^ NK cells have strong application prospects in clinical tumor treatment. On the one hand, researchers can enhance the practicality of NK cells by optimizing the source of therapeutic NK cells. On the other hand, enhancing NK cells' killing ability and persistence can improve the effectiveness of NK cell therapy.^223^, ^224^, ^225^ And EVs, as mediators of intercellular information exchange, can transmit circRNAs to NK cells, which affect tumor development by regulating the function of NK cells. For example, the exosomal circUHRF1 also originates from HCC cells, which mainly inhibits NK cell secretion of IFN‐γ and TNF‐α, and induces anti‐PD1 resistance by decreasing miR‐449c‐5p and increasing TIM‐3 expression.^226^ In summary, exosomal circRNAs can affect tumor progression by modulating the biological functions of NK cells, potentially offering a novel approach for tumor therapy.

### 5.7 Exosomal circRNAs and MDSCs

Bone marrow‐derived suppressor cells (MDSCs) undergo extensive expansion in the TME and can interfere with the killing effects of NK and T cells.^227^ MDSCs can directly activate the immune escape of tumor cells and indirectly increase tumor invasiveness.^228^ While prior research has identified a role for MDSCs in castration resistance, the involvement of MDSC exosomes in castration‐resistant prostate cancer remains uncertain. Researchers isolated MDSC exosomes and found that they can promote the proliferation and migration of PCa cells. Mechanistically, S100A9 in MDSC‐secreted exosomes activates circMID1 expression, promoting PCa progression via the miR‐506‐3p/MID1 axis.^229^, ^230^

Polymorphonuclear MDSCs (PMN‐MDSCs) have strong immunosuppressive effects and are activated in disease states.^231^ The presence of PMN‐MDSCs in the TME is a critical factor in immunotherapy failure and tumor progression, and is inversely related to patient prognosis. Among them, the presence of PMN‐MDSCs in the TME is one of the most important factors causing the failure of immunotherapy and intensified tumor progression, which is inversely related to patient prognosis. PMN‐MDSCs are activated by factors such as VEGF, M‐CSF, GM‐CSF, and IL‐6, and can upregulate the expression of ROS, iNOS, and Arg‐1, leading to tolerance to immunotherapy.^232^, ^233^ On the one hand, the exosomal circRNA_0013936 derived from bladder cancer increases the expression of FATP2 by targeting the miR‐320a/JAK2 axis. On the other hand, it also reduces RIPK3 levels by targeting the miR‐301b‐3p/CREB1 axis, thereby promoting the immunosuppressive function of PMN‐MDSCs and becoming one of the important reasons for the failure of immunotherapy.^234^

### 5.8 Exosomal circRNAs and neutrophils

Neutrophils, as prevalent innate immune cells, are crucial for immune regulation and infection defense.^235^ Tumor‐associated neutrophils (TANs) are the most important components of the TME, exhibiting a dual role in tumorigenesis.^236^ On the one hand, TANs activate tumor malignant progression by reshaping the extracellular matrix, promoting angiogenesis, driving immune suppression and tumor metastasis.^237^, ^238^ On the other hand, TANs can directly target tumor cells and perform antitumor functions. Similar to the naming for M1 and M2 macrophages, the N1 phenotype of neutrophils is an antitumor characteristic, while the N2 phenotype of neutrophils is a protumor characteristic.^239^, ^240^ Several exosomal circRNAs secreted by tumor cells can act on neutrophils and regulate their N1 to N2 transformation, which affects tumor growth and treatment. For example, recent studies indicate that exosomal circPACRGL originates from tumor cells and enhances the expression of downstream target TGF‐β1 by competitively binding to miR‐142‐3p and miR‐506‐3p. It subsequently promotes neutrophil polarization from N1 to N2, intensifying colorectal cancer growth and metastasis.^241^

### 5.9 Exosomal circRNAs and microbiota

Microorganisms are involved in the malignant progression of tumors, and about 20% of human tumors are closely related to microbiota.^242^, ^243^ The microbiota mainly promotes the development of cancer in three ways, including regulating tumor cell proliferation and apoptosis, guiding factor production and body metabolism, as well as altering the function of immune cells.^244^ In general, the body barrier can establish a symbiotic relationship with microorganisms. When the body's barrier is disrupted, the microbiota can activate inflammatory responses and immunosuppressive functions, leading to the transformation of normal tissues into tumors.^245^ And the microbiota can influence tumor development by modifying the expression of circRNAs in EVs. Researchers have found that the infection with *fusobacterium nucleatum* can induce the synthesis of hnRNP L‐dependent hsa_circ_0004085 and can also promote the encapsulation of hsa_circ_0004085 into EVs by regulating protein hnRNP A1. Subsequently, the exosomal hsa_circ_0004085 is delivered to the receptor cells, inhibiting ER stress and mediating resistance to 5‐fluorouracil/oxaliplatin by targeting the GRP78/ATF6p50 axis.^23^ Although the microbiota contributes to the synthesis of exosomal circRNAs and holds promise as a novel target for cancer therapy, research in this area remains limited and requires further investigation (Table 3).

**TABLE 3:** Exosomal circRNAs in the tumor microenvironment.

Exosomal circRNAs	Cancer type	Expression	Parent cell	Receptor cell	Genes and pathways	References
hsa_circ_0004085	Colorectal cancer	Up	Tumor cells	Tumor cells	GRP78/ATF6p50	23
circNEIL3	Glioma	Up	Tumor cells	Tumor‐associated macrophages	IGF2BP3	74
circTBPL1	Breast cancer	Up	CAFs	Tumor cells	miR‐653‐5p/TPBG	178
circDennd1b	Pituitary adenomas	Up	CAFs	Tumor cells	miR‐145‐5p/ ONECUT2	179
circ_0067557	Colorectal cancer	Up	CAFs	Tumor cells	Lin28	180
hsa_circ_0000437	Gastric cancer	Up	Tumor cells	Lymphatic endothelial cells	HSPA2–ERK signal	187
circRNA‐002178	Lung adenocarcinoma	Up	Tumor cells	T cells	miR‐34/PD‐L1	193
circUSP7	NSCLC	Up	Tumor cells	T cells	miR‐934/SHP2	194
hsa_circ_0000190	NSCLC	Up	Tumor cells	T cells	sPD‐L1	195
circCCAR1	HCC	Up	Tumor cells	T cells	miR‐127‐5p/WTAP	196
circTMEM181	HCC	Up	Tumor cells	T cells	miR‐488‐3p/CD39	197
circTRPS1	Bladder cancer	Up	Tumor cells	T cells	miR‐141‐3p/GLS1	198
circGSE1	HCC	Up	Tumor cells	Treg cells	miR‐324‐5p/ TGFBR1/Smad3	202
has_circ_0069313	OSCC	Up	Tumor cells	Treg cells	miR‐325‐3p/Foxp3	203
circTMCO3	Ovarian cancer	Up	M2 macrophages	Tumor cells	miR‐515‐5p/ITGA8	208
circFUT8	HCC	Down	M1 macrophages	Tumor cells	miR‐552‐3p/CHMP4B	209
circ_0001715	Lung cancer	Up	M0 macrophages	Tumor cells	miR‐205‐5p/TREM2	210
hsa_circ_0004658	HCC	Up	Macrophages activated by RBPJ	Tumor cells	miR‐499b‐5p/JAM3	211
circBTG2	Glioma	Up	Macrophages activated by RBPJ	Tumor cells	miR‐25‐3p/PTEN	212
circATP8A1	Gastric cancer	Up	Tumor cells	Macrophages	miR‐1‐3p/STAT6	213
circASPH	Colorectal cancer	Up	Tumor cells	Macrophages	IGF2BP2/STING	214
cSERPINE2	Breast cancer	Up	Tumor cells	Tumor‐associated macrophages	IL‐6	215
circPOLQ	Colorectal cancer	Up	Tumor cells	Macrophages	miR‐379‐3p/IL‐10 and STAT3 signal	215
circGLIS3	Gastric cancer	Up	Tumor cells	Macrophages	miR‐1343‐3p/PGK1; VIMENTIN protein	217
circ_0001142	Breast cancer	Up	Tumor cells	Macrophages	miR‐361‐3p/PIK3CB	218
circPLEKHM1	NSCLC	Up	Tumor cells	Macrophages	PAPC1 and eIF4G	219
circHIPK3	Lung cancer	Up	Tumor cells	Macrophages	/	220
circPTK2	Lung cancer	Up	Tumor cells	Macrophages	/	220
circUHRF1	HCC	Up	Tumor cells	NK cells	miR‐449c‐5p/TIM3	226
circMID1	Prostate cancer	Up	MDSCs	Tumor cells	miR‐506‐3p/MID1	229
circRNA_0013936	Bladder cancer	Up	Tumor cells	PMN‐MDSCs	miR‐320a/JAK2 axis; miR‐301b‐3p/CREB1	234
circPACRGL	Colorectal cancer	Up	Tumor cells	Neutrophil	miR‐142‐3p/miR‐506‐3p/TGF‐β1	241

## 6 POTENTIAL CLINICAL APPLICATION OF EXOSOMAL circRNAs

**FIGURE 5:**
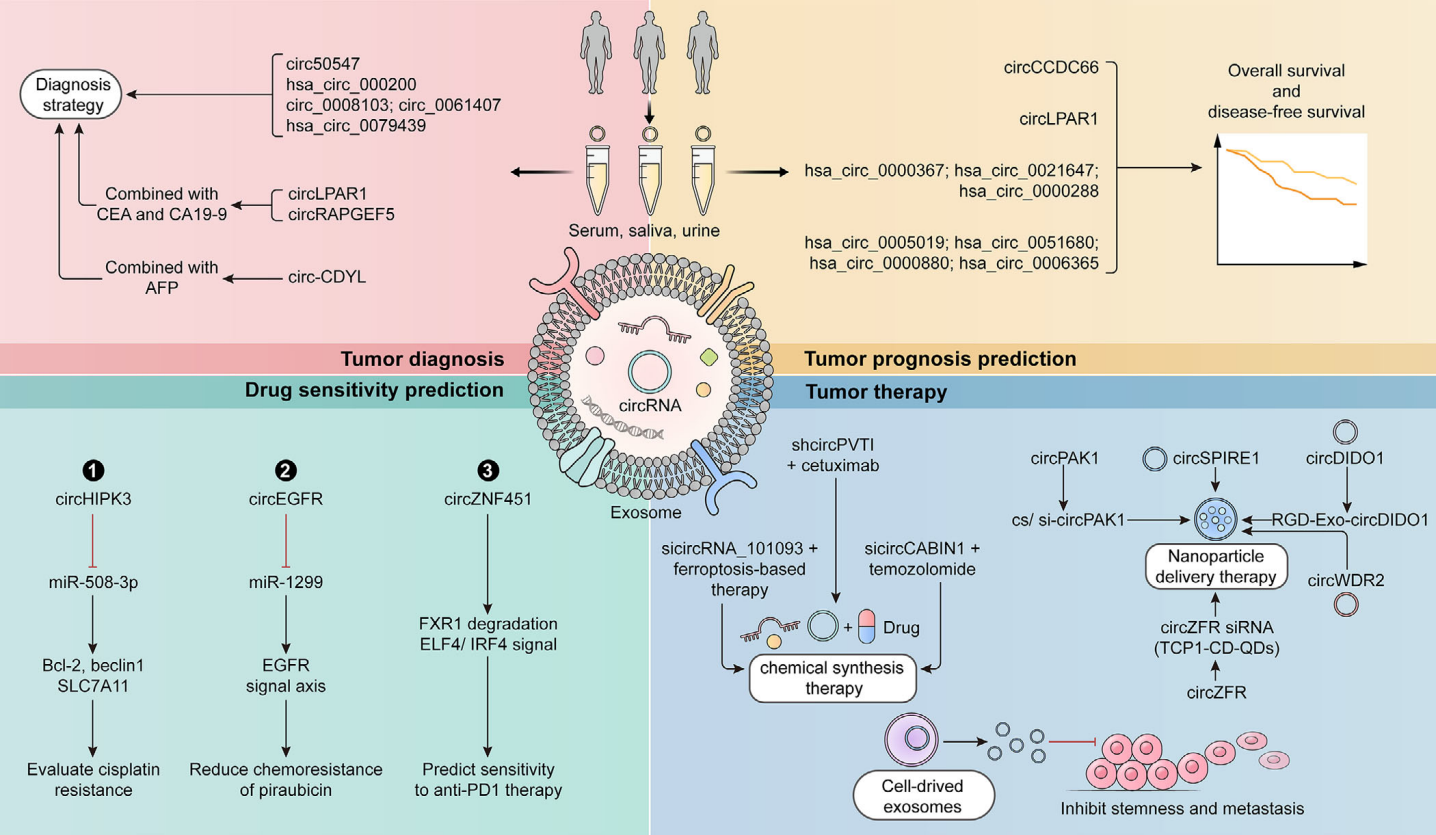
The clinical application strategies of exosomal circRNAs. Exosomal circRNAs can be regarded as biomarkers for cancer diagnosis, prognosis prediction and drug efficacy prediction. Meanwhile, exosomal circRNAs are promising therapeutic targets, significantly impacting chemical synthesis and nanoparticle delivery therapies.

Exosomal circRNAs affect cancer progression through the modulation of various signaling pathways and their associated downstream target proteins. Meanwhile, exosomal circRNAs can transfer and share information between different cells, which makes them extremely useful in the diagnosis, prognostic prediction and therapy of cancer. And the three primary antitumor approaches to targeting exosomal circRNAs include chemical synthesis of siRNA/shRNA (short hairpin RNA), designing nanomedicines, and directly using exosomes originating from various cells (Figure 5).

### 6.1 Diagnosis

Numerous scientific studies indicate that exosomal circRNAs hold significant potential as clinical diagnostic biomarkers for cancer patients in both tissue and blood samples.^67^ Circ50547, found in both tissue and serum exosomes of GC patients, enhances HNF1B expression and promotes GC progression by sequestering miR‐217. And exosomal circ50547 has a higher diagnostic value compared with serum circ50547 in GC.^246^ Moreover, exosomal hsa_circ_000200 binds to miR‐4659a/b‐3p and upregulates HBEGF levels to promote the activity of the TGF‐β/Smad signaling pathway, thereby exacerbating GC growth. Hsa_circ_000200 in EVs demonstrates superior diagnostic efficacy compared with tissues and serum, indicating its potential as a biomarker for GC diagnosis.^247^ Circ_0008103 and circ_0061407 in EVs are upregulated in NSCLC, enhancing tumor growth and invasion. And the downregulation of circ_0008103 and circ_0061407 in serum exosomes can reflect the stage and metastasis of tumors, providing new marker genes for the diagnosis of lung cancer.^248^ Researchers have found that the level of hsa_circ_0079439 in the exosomes of GC patients is significantly increased compared with normal individuals. In addition, the area under the curve (AUC) for hsa_circ_0079439 (with a value of 0.8595) significantly exceeded those of traditional cancer biomarkers, including CEA (0.5862), CA19‐9 (0.5660), alpha‐fetoprotein (0.5082), CA72‐4 (0.5360), and CA125 (0.5018). Substantially, the exosomal hsa_circ_0079439 derived from plasma shows potential as a biomarker for both early and late diagnosis of GC.^249^

A few exosomal circRNAs can be combined with standard clinical diagnostic markers (including CEA, CA19‐9, AFP, etc.), which further improves the reliability of diagnosis.^250^, ^251^ CircLPAR1 inhibits the direct effects of METTL3 and eIF3 h, reduces the expression of the oncogenic gene BRD4, and can be secreted into EVs, serving as a biomarker for colorectal cancer diagnosis. Research has shown that combining exosomal circLPAR1 with biomarkers CEA and CA19‐9 significantly enhances the diagnostic value for colorectal cancer.^252^ CircRAPGEF5 was detected in the exosomes of LUAD and was found to promote the expression of ZEB1, enhancing the invasion process by directly binding to miR‐1236‐3p. In addition, researchers also found that the combined detection of serum CEA and serum exosomal circRAPGEF5 can significantly improve the effectiveness of diagnosis, providing a new strategy for minimally invasive diagnosis.^253^ Moreover, researchers detected and analyzed the tissues and plasma exosomes of 30 healthy individuals, 30 chronic gastritis patients, and 64 GC patients, and identified circLPAR1, which is negatively correlated with GC progression. The AUC values of plasma exosomal circLPAR1, CA19‐9, and CEA in the diagnosis of GC were 0.836, 0.767, and 0.746, respectively, which were significantly lower than the AUC values of combined diagnosis (0.914). And the exosomal circLPAR1 is significantly associated with the survival of GC patients and holds substantial clinical value for diagnosis and prognostic assessment.^254^ Additionally, circ‐CDYL levels are notably elevated in the plasma exosomes of early liver cancer patients, dependent on hnRNPA2/B1‐mediated sorting for exosomal entry. The combination of exosomal circ‐CDYL and plasma protein AFP can effectively diagnose liver cancer (the value of AUC is 0.896), providing a promising diagnostic strategy for liver cancer.^255^

### 6.2 Prognostic prediction

A considerable portion of exosomal circRNAs not only play an important role in tumor diagnosis, but also have practical value in predicting the prognosis of tumor patients.^256^ In patients with pituitary adenoma, the expression of circCCDC66 in serum exosome shows a significant increase compared with the healthy population. Importantly, patients with lower circCCDC66 levels in serum exosomes exhibit longer disease‐free survival, indicating its potential as a biomarker for diagnosing and predicting the prognosis of pituitary adenomas.^257^ Deep sequencing and analysis of exosomes from high‐grade astrocytoma cells revealed that circRNAs in serum exosomes, such as hsa_circ_0003828, hsa_circ_0075828, and hsa_circ_0002976, can act as clinical biomarkers for blood diagnosis. Furthermore, hsa_circ_0005019, hsa_circ_0051680, hsa_circ_0000880, and hsa_circ_0006365 show promise as biomarkers for prognostic monitoring.^258^ Recent studies have found a close correlation between circHNRNPU and poor prognosis in patients with multiple myeloma. The exosomal circHNRNPU can encode the circHNRNPU_603aa protein, stabilize the expression of c‐Myc, and regulate the bone marrow microenvironment, which has the potential to become a new diagnostic and prognostic strategy for multiple myeloma.^259^ Moreover, microarray analysis of serum and bile samples identified hsa_circ_0000367, hsa_circ_0021647, and hsa_circ_0000288 are closely associated with the invasive phenotype of cholangiocarcinoma, suggesting their potential as diagnostic and prognostic markers superior to traditional markers.^260^

### 6.3 Drug sensitivity prediction

Some exosomal circRNAs can predict the effectiveness of chemotherapy and immunotherapy drugs.^261^, ^262^ The inhibition of exosomal circHIPK3 can regulate the expression of Bcl‐2, beclin1, and SLC7A11 by absorbing miR‐508‐3p, and then regulate the process of autophagy‐dependent ferroptosis and cisplatin resistance in GC. Further research indicates that exosomal circHIPK3 in serum is crucial for assessing cisplatin resistance.^263^ Also, the expression of circEGFR increased in tissues and plasma exosomes of TNBC patients and was negatively correlated with the poor prognosis of patients. CircEGFR promotes the growth, invasion, and EMT process by regulating the miR‐1299/EGFR signal axis, while reducing the chemosensitivity of pirarubicin in TNBC. And circEGFR in plasma exosomes shows potential as a clinical marker for diagnosing and predicting the prognosis of TNBC.^264^ In addition, exosomal circZNF451 enhances macrophage polarization and reduces cytotoxic CD8^+^ T cells by promoting FXR1 degradation and activating the ELF4/IRF4 signaling pathway in macrophages. Also, the exosomal circZNF451 can modify the immune microenvironment in LUAD and may serve as a biomarker for predicting sensitivity to anti‐PD1 therapy.^265^ In a word, exosomal circRNAs are pivotal in clinical diagnosis, prognosis and drug efficacy prediction, offering significant clinical value in carcinogenesis.

### 6.4 Tumor therapy

Numerous studies indicate that exosomal circRNAs regulate tumor progression through various molecular mechanisms, making them promising therapeutic targets.^266^, ^267^ CircPABPC1 is upregulated in colorectal cancer tissue and tumor cell exosomes, enhancing transcription in the nucleus by recruiting KDM4C to the HMGA2 promoter and decreasing H3K9me3 modification. Meanwhile, circPABPC1 in the cytoplasm regulates the expression of miR‐874/miR‐1292, increasing the expression of important proteins BMP4/ADAM19 in colorectal cancer metastasis and playing a role in antitumor metastasis therapy.^268^ Also, circRHOBTB3 is sorted into exosomes with the participation of protein SNF8 and slows down the progression of colorectal cancer by regulating metabolic signaling pathways and the production of ROS. Targeting circRHOBTB3 and elements for exosomal sorting will become a new strategy for responding to tumors.^269^

Targeting exosomal circRNAs in nanomedicine design can enhance tumor treatment efficacy and mitigate chemoresistance progression.^270^, ^271^, ^272^ Recently, researchers have used xenograft models to screen for circSPIRE1, which plays a potential role in tumor metastasis. The exosomal circSPIRE1 acts as a tumor suppressor by inhibiting angiogenesis and metastasis through upregulation of GALNT3 (enhancing glycosylation of E‐cadherin) and QKI protein (promoting expression of circSPIRE1). And nanomedicines synthesized from the circSPIRE1 plasmid can alleviate the metastasis of renal cell carcinoma and provide new targets for the treatment of metastatic cancer.^273^ CircZFR levels rise in colorectal cancer tissues and blood exosomes, correlating with cancer progression and metastasis. Mechanistically, circZFR targets miR‐3127‐5p both in vivo and in vitro to elevate RTKN2 levels and promote tumor growth. Moreover, researchers utilized TCP1‐CD‐QDs nanoparticles to load si‐circZFR (circZFR siRNA) and deliver it to the tumor area, which inhibited tumor growth in the patient‐derived xenograft (PDX) model.^274^ Additionally, researchers have demonstrated that RGD‐modified circDIDO1 packaged into exosomes (RGD–Exo–circDIDO1) inhibits the progression of GC in vivo and in vitro experiments. Mechanistically, RGD–Exo–circDIDO1 targets miR‐1307‐3p/SOSC2 signal in the treatment of GC, presenting a novel nanomedicine option for therapy.^275^ CircWDR62 is upregulated in temozolomide (TMZ)‐resistant glioma cells and exosomes compared with the control, closely related to the progression of TMZ resistance. Functionally, circWDR62 regulates downstream target genes MGMT and tumor progression by competitively binding to miR‐370‐3p. At the same time, using EVs to deliver circWDR62 can promote TMZ resistance and tumor malignant progression.^276^ Moreover, circPAK1 binds to 14–3‐3 ζ and reduces its recruitment of YAP, thereby increasing the nuclear localization of YAP, inducing Hippo activity, and promoting the progression of HCC. Researchers have also found that using CS/si‐circPAK1 nanocomposites could better inhibit tumor growth and migration. In addition, the expression of circPAK1 in EVs induces the emergence of lenvatinib resistance, suggesting it as a potential target for HCC treatment.^277^

The combination of siRNA/shRNA targeting exosomal circRNAs and tumor therapeutic drugs can enhance antitumor effects.^278^ CircPVT1 is secreted by laryngeal cancer cells into exosomes and enters vascular epithelial cells. It then induces angiogenesis by absorbing miR‐30c‐5p, increasing Rap1b levels and upregulating VEGFR2 and PI3K/AKT signaling pathways. Meanwhile, researchers have found that the combination of circPVT1 shRNA and cetuximab in the PDX model can effectively inhibit vascularization and laryngeal cancer progression.^279^ And the exosomal circCABIN1 secreted by TMZ‐resistant cells acts on receptor cells and activates the ErbB signaling pathway by targeting the miR‐637/OLFML3 signaling axis, ultimately leading to the exacerbation of TMZ resistance. In a mouse xenograft model, the use of engineered exosomes targeting circCABIN1 and OLFML3 significantly enhances the therapeutic efficacy of TMZ, indicating their potential as a tool for clinical cancer therapy.^280^ In LUAD, the exosomal circRNA_101093 can maintain the level of circRNA within tumor cells and reduce cell sensitivity to ferroptosis by interacting with FABP3. And in preclinical research models, it was found that reducing exosomal circRNA_101093 can enhance the effectiveness of ferroptosis‐based therapy.^281^

The exosomal circRNAs secreted by tumor cells can usually exert immunosuppressive functions and promote immune escape.^282^ And exosomal circRNAs in the TME can dysregulate immune cells, such as CD8^+^ T cells, NK cells and dendritic cells, influencing immune checkpoint therapy. In patients with ICC, the levels of exosomal circ‐PTPN22 and circ‐ADAMTS6 in plasma are significantly elevated, contributing to T cell depletion and inducing neutrophil extracellular trap formation. Exosomal circ‐PTPN22 and circ‐ADAMTS6 could serve as novel targets for liquid biopsy and contribute to immune checkpoint blockade therapy.^283^ In addition, exosomal circ_001264, derived from acute myeloid leukemia (AML) cells, activates the p38/STAT3 signaling axis by regulating RAF1 levels, followed by activating macrophage polarization toward the M2 type and elevating PD‐L1 expression. At the same time, the combination of circ_001264 siRNA and aPD‐L1 showed significant antitumor effects in AML.^284^

Some cancers, such as breast cancer, are closely related to obesity. Adipose‐derived exosomal circCRIM1 exacerbates breast cancer progression by downregulating miR‐503‐5p and activating the OGA/FBP1 signaling pathway. Therefore, targeting the adipose‐associated exosomal circCRIM1 is a promising strategy to alleviate breast cancer.^285^ Moreover, researchers have discovered that circ_0037104 in Hu‐MSC‐derived (human umbilical cord‐derived mesenchymal stem cells) exosomes primarily binds to miR‐620, elevates APAF1 levels, and suppresses the stemness and metastasis of cholangiocarcinoma, offering novel antitumor therapeutic strategies.^286^

Recently, researchers from Shanghai Jiao Tong University conducted clinical trials on the circRNA drug HM2002 injection. They administered HM2002 via epicardial myocardial injection in patients with ischemic heart failure undergoing Coronary Artery Bypass Grafting, in order to evaluate the safety and efficacy of HM2002. However, further clinical trials of circRNA drugs in tumor therapy are still needed. In the future, researchers can achieve effective delivery and expression of circRNAs in tumor tissues by developing new delivery strategies. In addition, circRNA may also be combined with other therapeutic strategies, such as targeted therapy and immunotherapy, to jointly combat the malignant progression of cancer and enhance treatment efficacy. In summary, we have summarized some exosomal circRNAs that play critical roles in tumorigenesis and may provide promising new strategies for cancer diagnosis and treatment (Table 4).

**TABLE 4:** The potential clinical application of exosomal circRNAs in various tumors.

Application	Exosomal circRNAs	Cancer type	Expression	Function	References
Diagnosis	circ50547	Gastric cancer	Up	Diagnostic biomarkers	246
	hsa_circ_000200	Gastric cancer	Up	Diagnostic biomarkers	247
	circ_0008103; circ_0061407	NSCLC	Up	Diagnostic biomarkers	248
	hsa_circ_0079439	Gastric cancer	Up	Biomarkers for the early and late diagnosis	249
	circRAPGEF5	Lung adenocarcinoma	Up	Minimally invasive diagnosis	253
	circ‐CDYL	HCC	Up	Diagnostic biomarkers	255
	hsa_circ_0003828; hsa_circ_0075828; hsa_circ_0002976	High‐grade astrocytoma	Up	Diagnostic biomarkers	258
Prognosis prediction	circLPAR1	Colorectal cancer; gastric cancer	Down	Diagnostic biomarkers; prognostic biomarkers	252
	circCCDC66	Pituitary adenoma	Up	Diagnostic biomarkers; prognostic biomarkers	257
	hsa_circ_0005019; hsa_circ_0051680; hsa_circ_0000880; hsa_circ_0006365	High‐grade astrocytoma	Up	Prognostic biomarkers	258
	circHNRNPU	Multiple myeloma	Up	Diagnostic biomarkers	259
	hsa_circ_0000367; hsa_circ_0021647; hsa_circ_0000288	Cholangiocarcinoma	Up	Diagnostic biomarkers; prognostic biomarkers	260
Chemoresistance prediction	circHIPK3	Gastric cancer	Up	Evaluate cisplatin resistance	263
	circEGFR	TNBC	Up	Reduce pirarubicin sensitivity	264
	circZNF451	Lung cancer	Up	Predict sensitivity to anti‐PD1	265
Therapeutic target	circPABPC1	Colorectal cancer	Up	Therapeutic targets for tumor metastasis	268
	circRHOBTB3	Colorectal cancer	Down	Inhibit tumor progression	269
	circSPIRE1	Renal cell carcinoma	Down	Alleviate the metastasis	273
	circZFR	Colorectal cancer	Up	Promote metastasis	274
	circDIDO1	Gastric cancer	Down	Inhibit tumor progression	275
	circWDR62	Glioma	Up	Promote temozolomide resistance	276
	circPAK1	HCC	Up	Induce lenvatinib resistance	277
	circPVT1	Laryngeal cancer	Up	Induces angiogenesis	279
	circCABIN1	Glioblastoma	Up	Promote temozolomide resistance	280
	circRNA_101093	Lung adenocarcinoma	Up	Reduce sensitivity to ferroptosis	281
	circ‐PTPN22; circ‐ADAMTS6	ICC	Up	Induce resistance to immune checkpoint therapy	283
	circ_001264	Acute myeloid leukemia	Up	Activate M2 macrophages	284
	circCRIM1	Breast cancer	Up	Promote tumor growth	285
	circ_0037104	Cholangiocarcinoma	/	Inhibit the stemness and metastasis	286

## 7 CONCLUSION AND PERSPECTIVES

Recent studies have shown that exosomal circRNAs play an important role in tumorigenesis and progression. Exosomal circRNAs can affect the malignant progression of tumors by regulating various biological processes including proliferation, metastasis, autophagy, angiogenesis, drug resistance, metabolism, and immune escape. And exosomal circRNAs regulate tumor development through various molecular mechanisms. Certain exosomal circRNAs translate into small peptides that regulate tumor‐related gene expression, while others bind directly to target miRNAs and proteins, influencing tumor‐related signaling pathways. For example, exosomal circRNAs (including hsa_circ_0010467, circSYT15, circDCAF8, and circBACH1) can promote the acquisition of chemoresistance in tumor cells by targeting miRNAs regulation, while others such as circRNA–CREIT and circRABL2B enhance the sensitivity of chemotherapy by downregulating proteins and resistance‐related signaling pathways. Numerous studies indicate that exosomal circRNAs contribute to tumor malignancy by encoding novel peptides. Exosomal circATG4B and circHNRNPU regulate drug resistance and TME by encoding proteins circATG4B‐222aa and circHNRNPU_603aa, respectively. Not all circRNAs are sorted into exosomes, but existing research has shown that a few circRNAs may have special sequence structures or bind to sorting‐related proteins that allow them to be transported to other cells through exosomes, thereby regulating the growth and migration of tumor cells. By delving deeper into the function of circRNAs in cancer progression, researchers can design inhibitors or interfering RNAs targeting these circRNAs to slow down tumor proliferation and spread. Furthermore, due to the function of exosomes in transmitting biological regulatory molecules between cells, researchers can use exosomes to transport circRNA to tumor cells, thereby reversing cancer progression, improving targeted therapy efficacy and reducing side effects. Despite numerous studies on the role and mechanism of circRNAs in tumor progression, the molecular mechanism of their entry into exosomes remains unclear. We hope that researchers can elucidate the molecular mechanism by which circRNAs are sorted into exosomes and the sequence characteristics of circRNAs that can enter exosomes in the future.

With the development of single‐cell sequencing technology, high‐throughput sequencing technology, and various bioinformatics algorithms, research on circRNAs has made great progress. RNA‐seq, a high‐throughput sequencing technology, is widely used to comprehensively detect and identify circRNA expression profiles in cells and tissues.^287^ In recent years, the increasing sensitivity and accuracy of RNA‐seq have provided technical support for the identification of circRNAs. Single‐cell sequencing technology uncovers dynamic circRNA changes across developmental stages and disease states at the single‐cell level, offering new insights into circRNA functions.^288^ In addition, advancements in bioinformatics have led to the development of efficient circRNA prediction algorithms, including find_circ, circRNA_finder, CIRI, CIRCexplorer, and MapSplice.^289^ Some emerging analytical methods like machine learning and deep learning can also explore the relationship between circRNAs and cancer progression, offering new strategies for their clinical application of circRNAs. In addition to the advancement of sequencing technology, numerous studies are investigating the molecular functions and mechanisms of circRNAs using PDX tumor mouse models and tumor organoid models. However, the current research is mainly based on cellular and animal experiments, and there is still a long way before achieving clinical application translation. Additional research is required to confirm these findings and establish practical clinical protocols.

Exosomal circRNAs in the tumor immune microenvironment modulate tumor growth by acting on various types of cells, including CAFs, endothelial cells, T cells, Treg cells, macrophages, neutrophils, NK cells, and microbiota. Exosomal circRNAs derived from tumor cells can be absorbed by immune cells and affect tumor immune escape by activating downstream tumor‐related genes. Moreover, exosomal circRNAs secreted by immune cells and fibroblasts can be transmitted to tumor cells, regulating their growth, metastasis, and drug resistance. For example, exosomal hsa_circ_0004658 and circBTG2 derived from activated macrophages can inhibit tumor growth in liver cancer and glioma, respectively. Also, wang et al. collected plasma exosomes from healthy individuals and ICC patients for transcriptome sequencing, identifying circ‐PTPN22 and circ‐ADAMTS6 as potential biomarkers through differential gene analysis.^283^ And targeted inhibition of exosomal circ‐PTPN22 and circ‐ADAMTS6 can mitigate tumor progression by decreasing T cell exhaustion and neutrophil extracellular trap formation. A few exosomal circRNAs like circ_001264 can enhance antitumor effects when their siRNA is used in combination with aPD‐L1. In addition, some harmful bacteria can activate the expression of exosomal circRNAs and stimulate tumor growth by transmitting genetic information between tumor cells. However, there is still limited research on the role of other types of cells, apart from T cells and macrophages, in regulating tumor progression through exosomal circRNAs. Also, the relationship between probiotics and exosomal circRNAs in carcinogenesis is still unknown.

Exosomal circRNAs have great clinical application prospects. Exosomal circRNA, known for its high abundance and stability, is found in various body fluids like blood and urine, making it an ideal diagnostic and prognostic biomarker. Researchers have identified some exosomal circRNAs as potential biomarkers for cancer diagnosis, and their combination with traditional biomarkers (such as CA19‐9 and CEA) can enhance diagnostic accuracy. For instance, exosomal circ50547, hsa_circ_0079439, circ_0008103, and circ_0061407 are linked to cancer progression and survival, indicating their potential to serve as biomarkers for cancer diagnosis. Exosomal circLPAR1, circRAPGEF5, and circ‐CDYL can be combined with traditional markers to improve diagnostic accuracy. The level of exosomal circRNAs may be closely associated with the prognosis of patients. Researchers can assess disease progression and therapy efficacy by monitoring changes in exosomal circRNAs, and develop personalized therapy plans for patients. Through in‐depth research on exosomal circRNAs, early signals of tumor occurrence can be revealed, providing new methods for tumor prevention and a scientific basis for the formulation of public health policies.

Functional circRNAs may serve as future therapeutic targets for cancer. Researchers can target circRNAs for tumor therapy using ASOs, siRNA, and CRISPR‐Cas13a. Researchers can also reverse the drug resistance of tumor cells by regulating the synthesis and secretion of exosomes, as well as affecting the uptake of target cells. To achieve precise treatment of tumors, the combination of various exosomal circRNAs with other noncoding RNAs, proteins, and DNAs has become a new possibility. In addition, the development of nanomedicines targeting exosomal circRNAs has the potential to become a new strategy for tumor therapy. However, this method faces extensive limitations, including the loading efficiency of circRNAs, the tumor‐targeting ability of nanoparticles, and immunogenicity. In order to improve these shortcomings, researchers have also attempted to develop exosomal circRNAs derived from immune cells for tumor therapy, which has become a promising therapeutic strategy.

Currently, there is still much debate about the application of exosomal circRNAs in the clinical therapy of tumors. Despite the stable structures and high abundance of exosomal circRNAs in various body fluids, some researchers argue that false positives may occur in their identification, posing a challenge to their standardization as diagnostic markers. And exosomes, as one of the natural nanocarriers, have the advantages of low toxicity and high targeting. However, the delivery efficiency of exosomal circRNA remains one of the current controversies. Although exosomal circRNAs have made some progress in clinical diagnosis and therapy, more research is still needed for their clinical application.

## AUTHOR CONTRIBUTIONS

Junshu Li, Wencheng Zhou, and Huiling Wang drafted the manuscript. Hongxin Deng and Meijuan Huang designed and revised the manuscript. All authors have reviewed and endorsed the final manuscript.

## CONFLICT OF INTEREST STATEMENT

The authors declare that they have no conflict of interest.

## ETHICS STATEMENT

Not applicable.

## Data Availability

Not applicable.

## References

[1] Cao M , Li H , Sun D , et al. Cancer burden of major cancers in China: a need for sustainable actions. Cancer Commun. 2020;40(5):205‐210. 10.1002/cac2.12025 PMC7667573 32359212

[2] Bray F , Laversanne M , Sung H , et al. Global cancer statistics 2022: GLOBOCAN estimates of incidence and mortality worldwide for 36 cancers in 185 countries. CA Cancer J Clin. 2024;74(3):229‐263. 38572751 10.3322/caac.21834

[3] Siegel RL , Giaquinto AN , Jemal A . Cancer statistics, 2024. CA Cancer J Clin. 2024;74(1):12‐49. 38230766 10.3322/caac.21820

[4] Schafer EJ , Jemal A , Wiese D , et al. Disparities and trends in genitourinary cancer incidence and mortality in the USA. Eur Urol. 2023;84(1):117‐126. 36566154 10.1016/j.eururo.2022.11.023

[5] Boehmer U , Chang S , Sanchez NF , et al. Cancer survivors' health behaviors and outcomes: a population‐based study of sexual and gender minorities. J Natl Cancer Inst. 2023;115(10):1164‐1170. 37421397 10.1093/jnci/djad131 PMC10560602

[6] Shelton J , Zotow E , Smith L , et al. 25 year trends in cancer incidence and mortality among adults aged 35–69 years in the UK, 1993–2018: retrospective secondary analysis. Bmj. 2024;384. 10.1136/bmj-2023-076962 PMC10935512 38479774

[7] Lin Z , Ji Y , Zhou J , et al. Exosomal circRNAs in cancer: implications for therapy resistance and biomarkers. Cancer Lett. 2023:216245. 37247772 10.1016/j.canlet.2023.216245

[8] Lauwers E , Wang Y‐C , Gallardo R , et al. Hsp90 mediates membrane deformation and exosome release. Mol Cell. 2018;71(5):689‐702.e9. 30193096 10.1016/j.molcel.2018.07.016

[9] Deng J , Liao S , Chen C , et al. Specific intracellular retention of circSKA3 promotes colorectal cancer metastasis by attenuating ubiquitination and degradation of SLUG. Cell Death Dis. 2023;14(11):750. 37973787 10.1038/s41419-023-06279-w PMC10654574

[10] Du WW , Yang W , Li X , et al. The circular RNA circSKA3 binds integrin β1 to induce invadopodium formation enhancing breast cancer invasion. Mol Ther. 2020;28(5):1287‐1298. 32229309 10.1016/j.ymthe.2020.03.002 PMC7210749

[11] Liu Q‐W , He Y , Xu WW . Molecular functions and therapeutic applications of exosomal noncoding RNAs in cancer. Exp Mol Med. 2022;54(3):216‐225. 35352001 10.1038/s12276-022-00744-w PMC8980040

[12] Pisignano G , Michael DC , Visal TH , et al. Going circular: history, present, and future of circRNAs in cancer. Oncogene. 2023;42(38):2783‐2800. 37587333 10.1038/s41388-023-02780-w PMC10504067

[13] Lin H , Wang Y , Wang P , et al. Mutual regulation between N6‐methyladenosine (m6A) modification and circular RNAs in cancer: impacts on therapeutic resistance. Mol Cancer. 2022;21(1):148. 35843942 10.1186/s12943-022-01620-x PMC9290271

[14] Li J , Zhang G , Liu C‐G , et al. The potential role of exosomal circRNAs in the tumor microenvironment: insights into cancer diagnosis and therapy. Theranostics. 2022;12(1):87. 34987636 10.7150/thno.64096 PMC8690929

[15] Xu H‐Z , Lin X‐Y , Xu Y‐X , et al. An emerging research: the role of hepatocellular carcinoma‐derived exosomal circRNAs in the immune microenvironment. Front Immunol. 2023;14:1227150. 37753074 10.3389/fimmu.2023.1227150 PMC10518420

[16] De Visser KE , Joyce JA . The evolving tumor microenvironment: from cancer initiation to metastatic outgrowth. Cancer Cell. 2023;41(3):374‐403. 36917948 10.1016/j.ccell.2023.02.016

[17] Xu Z , Chen Y , Ma L , et al. Role of exosomal non‐coding RNAs from tumor cells and tumor‐associated macrophages in the tumor microenvironment. Mol Ther. 2022;30(10):3133‐3154. 35405312 10.1016/j.ymthe.2022.01.046 PMC9552915

[18] Zhang Y , Luo J , Yang W , et al. CircRNAs in colorectal cancer: potential biomarkers and therapeutic targets. Cell Death Dis. 2023;14(6):353. 37296107 10.1038/s41419-023-05881-2 PMC10250185

[19] Zhou W , Zhou Y , Chen X , et al. Pancreatic cancer‐targeting exosomes for enhancing immunotherapy and reprogramming tumor microenvironment. Biomaterials. 2021;268:120546. 33253966 10.1016/j.biomaterials.2020.120546

[20] Elahi FM , Farwell DG , Nolta JA , et al. Preclinical translation of exosomes derived from mesenchymal stem/stromal cells. Stem Cells. 2020;38(1):15‐21. 31381842 10.1002/stem.3061 PMC7004029

[21] Greening DW , Xu R , Ale A , et al. Extracellular vesicles as next generation immunotherapeutics. Semin Cancer Biol. 2023;90:73‐100. 36773820 10.1016/j.semcancer.2023.02.002

[22] Liu Z , Wang T , She Y , et al. N 6‐methyladenosine‐modified circIGF2BP3 inhibits CD8^+^ T‐cell responses to facilitate tumor immune evasion by promoting the deubiquitination of PD‐L1 in non‐small cell lung cancer. Mol Cancer. 2021;20:1‐25. 34416901 10.1186/s12943-021-01398-4 PMC8377850

[23] Hui B , Zhou C , Xu Y , et al. Exosomes secreted by Fusobacterium nucleatum‐infected colon cancer cells transmit resistance to oxaliplatin and 5‐FU by delivering hsa_circ_0004085. J Nanobiotechnol. 2024;22(1):62. 10.1186/s12951-024-02331-9 PMC10867993 38360615

[24] Yi Q , Yue J , Liu Y , et al. Recent advances of exosomal circRNAs in cancer and their potential clinical applications. J Transl Med. 2023;21(1):516. 37525158 10.1186/s12967-023-04348-4 PMC10388565

[25] Xian J , Su W , Liu L , et al. Identification of three circular RNA cargoes in serum exosomes as diagnostic biomarkers of non–small‐cell lung cancer in the chinese population. J Mol Diagn. 2020;22(8):1096‐1108. 32535085 10.1016/j.jmoldx.2020.05.011

[26] Li J , Zhang Y , Dong P‐Y , et al. A comprehensive review on the composition, biogenesis, purification, and multifunctional role of exosome as delivery vehicles for cancer therapy. Biomed Pharmacother. 2023;165:115087. 37392659 10.1016/j.biopha.2023.115087

[27] Xie H , Yao J , Wang Y , et al. Exosome‐transmitted circVMP1 facilitates the progression and cisplatin resistance of non‐small cell lung cancer by targeting miR‐524‐5p‐METTL3/SOX2 axis. Drug Deliv. 2022;29(1):1257‐1271. 35467477 10.1080/10717544.2022.2057617 PMC9045767

[28] Zeng W , Liu Y , Li WT , et al. CircFNDC3B sequestrates miR‐937‐5p to derepress TIMP3 and inhibit colorectal cancer progression. Mol Oncol. 2020;14(11):2960‐2984. 32896063 10.1002/1878-0261.12796 PMC7607164

[29] Wang X , Zhang H , Yang H , et al. Exosome‐delivered circRNA promotes glycolysis to induce chemoresistance through the miR‐122‐PKM2 axis in colorectal cancer. Mol Oncol. 2020;14(3):539‐555. 31901148 10.1002/1878-0261.12629 PMC7053238

[30] Cheng L , Hill AF . Therapeutically harnessing extracellular vesicles. Nat Rev Drug Discov. 2022;21(5):379‐399. 35236964 10.1038/s41573-022-00410-w

[31] Hendrix A , Lippens L , Pinheiro C , et al. Extracellular vesicle analysis. Nat Rev Method Prime. 2023;3(1):56. 10.1038/s43586-023-00240-z PMC12968065 41809514

[32] Kalluri R , LeBleu VS . The biology, function, and biomedical applications of exosomes. Science. 2020;367(6478):eaau6977. 32029601 10.1126/science.aau6977 PMC7717626

[33] Mashouri L , Yousefi H , Aref AR , et al. Exosomes: composition, biogenesis, and mechanisms in cancer metastasis and drug resistance. Mol Cancer. 2019;18:1‐14. 30940145 10.1186/s12943-019-0991-5 PMC6444571

[34] Tuo B , Chen Z , Dang Q , et al. Roles of exosomal circRNAs in tumour immunity and cancer progression. Cell Death Dis. 2022;13(6):539. 35676257 10.1038/s41419-022-04949-9 PMC9177590

[35] Yan H , Li Y , Cheng S , et al. Advances in analytical technologies for extracellular vesicles. Anal Chem. 2021;93(11):4739‐4774. 33635060 10.1021/acs.analchem.1c00693

[36] Dong J , Zeng Z , Sun R , et al. Specific and sensitive detection of CircRNA based on netlike hybridization chain reaction. Biosens Bioelectron. 2021;192:113508. 34284304 10.1016/j.bios.2021.113508

[37] Dong J , Zeng Z , Huang Y , et al. Challenges and opportunities for circRNA identification and delivery. Crit Rev Biochem Mol. 2023;58(1):19‐35. 10.1080/10409238.2023.2185764 36916323

[38] Lee JU , Kim WH , Lee HS , et al. Quantitative and specific detection of exosomal miRNAs for accurate diagnosis of breast cancer using a surface‐enhanced Raman scattering sensor based on plasmonic head‐flocked gold nanopillars. Small. 2019;15(17):1804968. 10.1002/smll.201804968 30828996

[39] Van Niel G , d'Angelo G , Raposo G . Shedding light on the cell biology of extracellular vesicles. Nat Rev Mol Cell Biol. 2018;19(4):213‐228. 29339798 10.1038/nrm.2017.125

[40] Marar C , Starich B , Wirtz D . Extracellular vesicles in immunomodulation and tumor progression. Nat Immunol. 2021;22(5):560‐570. 33753940 10.1038/s41590-021-00899-0 PMC9389600

[41] Maacha S , Bhat AA , Jimenez L , et al. Extracellular vesicles‐mediated intercellular communication: roles in the tumor microenvironment and anti‐cancer drug resistance. Mol Cancer. 2019;18:1‐16. 30925923 10.1186/s12943-019-0965-7 PMC6441157

[42] Aloi N , Drago G , Ruggieri S , et al. Extracellular vesicles and immunity: at the crossroads of cell communication. Int J Mol Sci. 2024;25(2):1205. 38256278 10.3390/ijms25021205 PMC10816988

[43] Stoorvogel W , Strous GJ , Geuze HJ , et al. Late endosomes derive from early endosomes by maturation. Cell. 1991;65(3):417‐427. 1850321 10.1016/0092-8674(91)90459-c

[44] Liu J , Ren L , Li S , et al. The biology, function, and applications of exosomes in cancer. Acta Pharm Sin B. 2021;11(9):2783‐2797. 34589397 10.1016/j.apsb.2021.01.001 PMC8463268

[45] Kogure A , Yoshioka Y , Ochiya T . Extracellular vesicles in cancer metastasis: potential as therapeutic targets and materials. Int J Mol Sci. 2020;21(12):4463. 32585976 10.3390/ijms21124463 PMC7352700

[46] Vu LT , Gong J , Pham TT , et al. MicroRNA exchange via extracellular vesicles in cancer. Cell Prolif. 2020;53(11):e12877. 33169503 10.1111/cpr.12877 PMC7653238

[47] Wang H‐X , Gires O . Tumor‐derived extracellular vesicles in breast cancer: from bench to bedside. Cancer Lett. 2019;460:54‐64. 31233837 10.1016/j.canlet.2019.06.012

[48] Sil S , Dagur RS , Liao K , et al. Strategies for the use of extracellular vesicles for the delivery of therapeutics. J Neuroimmune Pharm. 2020;15:422‐442. 10.1007/s11481-019-09873-y PMC7044028 31456107

[49] Eguchi T , Sheta M , Fujii M , et al. Cancer extracellular vesicles, tumoroid models, and tumor microenvironment. Semin Cancer Biol. 2022;86:112‐126. 35032650 10.1016/j.semcancer.2022.01.003

[50] Adnani L , Spinelli C , Tawil N , et al. Role of extracellular vesicles in cancer‐specific interactions between tumour cells and the vasculature. Semin Cancer Biol. 2022;87:196‐213. 36371024 10.1016/j.semcancer.2022.11.003

[51] Zhou E , Li Y , Wu F , et al. Circulating extracellular vesicles are effective biomarkers for predicting response to cancer therapy. EBioMedicine. 2021;67:103365. 33971402 10.1016/j.ebiom.2021.103365 PMC8121992

[52] Calvo V , Izquierdo M . T lymphocyte and CAR‐T cell‐derived extracellular vesicles and their applications in cancer therapy. Cells. 2022;11(5):790. 35269412 10.3390/cells11050790 PMC8909086

[53] Shi Y , Lu Y , You J . Antigen transfer and its effect on vaccine‐induced immune amplification and tolerance. Theranostics. 2022;12(13):5888. 35966588 10.7150/thno.75904 PMC9373810

[54] Zhang H , Xie Y , Li W , et al. CD4^+^ T cell‐released exosomes inhibit CD8^+^ cytotoxic T‐lymphocyte responses and antitumor immunity. Cell Mol Immunol. 2011;8(1):23‐30. 21200381 10.1038/cmi.2010.59 PMC4002994

[55] Martinez‐Usatorre A , De Palma M . Dendritic cell cross‐dressing and tumor immunity. EMBO Mol Med. 2022;14(10):e16523. 35959554 10.15252/emmm.202216523 PMC9549722

[56] Kristensen LS , Andersen MS , Stagsted LV , et al. The biogenesis, biology and characterization of circular RNAs. Nat Rev Genet. 2019;20(11):675‐691. 31395983 10.1038/s41576-019-0158-7

[57] Zhou W‐Y , Cai Z‐R , Liu J , et al. Circular RNA: metabolism, functions and interactions with proteins. Mol Cancer. 2020;19:1‐19. 33317550 10.1186/s12943-020-01286-3 PMC7734744

[58] Beilerli A , Gareev I , Beylerli O , et al. Circular RNAs as biomarkers and therapeutic targets in cancer. Semin Cancer Biol. 2022;83:242‐252. 33434640 10.1016/j.semcancer.2020.12.026

[59] Li F , Yang Q , He AT , et al. Circular RNAs in cancer: limitations in functional studies and diagnostic potential. Semin Cancer Biol. 2021;75:49‐61. 33035655 10.1016/j.semcancer.2020.10.002

[60] Xu X , Zhang J , Tian Y , et al. CircRNA inhibits DNA damage repair by interacting with host gene. Mol Cancer. 2020;19:1‐19. 32838810 10.1186/s12943-020-01246-x PMC7446195

[61] Conn VM , Gabryelska M , Toubia J , et al. Circular RNAs drive oncogenic chromosomal translocations within the MLL recombinome in leukemia. Cancer Cell. 2023;41(7):1309‐1326.e10. 37295428 10.1016/j.ccell.2023.05.002

[62] Conn VM , Chinnaiyan AM , Conn SJ . Circular RNA in cancer. Nat Rev Cancer. 2024:1‐17. 10.1038/s41568-024-00721-7 39075222

[63] Kristensen LS , Jakobsen T , Hager H , et al. The emerging roles of circRNAs in cancer and oncology. Nat Rev Clin Oncol. 2022;19(3):188‐206. 34912049 10.1038/s41571-021-00585-y

[64] Fu B , Liu W , Zhu C , et al. Circular RNA circBCBM1 promotes breast cancer brain metastasis by modulating miR‐125a/BRD4 axis. Int J of Biol Sci. 2021;17(12):3104. 34421353 10.7150/ijbs.58916 PMC8375234

[65] Li H , Jiao W , Song J , et al. circ‐hnRNPU inhibits NONO‐mediated c‐Myc transactivation and mRNA stabilization essential for glycosylation and cancer progression. J Exp Clin Cancer Res. 2023;42(1):313. 37993881 10.1186/s13046-023-02898-5 PMC10666356

[66] Chen C‐K , Cheng R , Demeter J , et al. Structured elements drive extensive circular RNA translation. Mol Cell. 2021;81(20):4300‐4318.e13. 34437836 10.1016/j.molcel.2021.07.042 PMC8567535

[67] Zhang F , Jiang J , Qian H , et al. Exosomal circRNA: emerging insights into cancer progression and clinical application potential. J Hematol Oncol. 2023;16(1):67. 37365670 10.1186/s13045-023-01452-2 PMC10294326

[68] Corrado C , Barreca MM , Zichittella C , et al. Molecular mediators of RNA loading into extracellular vesicles. Cells. 2021;10(12):3355. 34943863 10.3390/cells10123355 PMC8699260

[69] Teng Y , Ren Y , Hu X , et al. MVP‐mediated exosomal sorting of miR‐193a promotes colon cancer progression. Nat Commun. 2017;8(1):14448. 28211508 10.1038/ncomms14448 PMC5321731

[70] He T , Zhang Q , Xu P , et al. Extracellular vesicle‐circEHD2 promotes the progression of renal cell carcinoma by activating cancer‐associated fibroblasts. Mol Cancer. 2023;22(1):117. 37481520 10.1186/s12943-023-01824-9 PMC10362694

[71] Ghossoub R , Chéry M , Audebert S , et al. Tetraspanin‐6 negatively regulates exosome production. Proc Natl Acad Sci USA. 2020;117(11):5913‐5922. 32108028 10.1073/pnas.1922447117 PMC7084133

[72] Zhou X , Hong Y , Liu Y , et al. Intervening in hnRNPA2B1‐mediated exosomal transfer of tumor‐suppressive miR‐184‐3p for tumor microenvironment regulation and cancer therapy. J Nanobiotechnol. 2023;21(1):422. 10.1186/s12951-023-02190-w PMC10644646 37957722

[73] Yao Y , Chen C , Wang J , et al. Circular RNA circATP9A promotes non‐small cell lung cancer progression by interacting with HuR and by promoting extracellular vesicles‐mediated macrophage M2 polarization. J Exp Clin Cancer Res. 2023;42(1):330. 38049814 10.1186/s13046-023-02916-6 PMC10696866

[74] Pan Z , Zhao R , Li B , et al. EWSR1‐induced circNEIL3 promotes glioma progression and exosome‐mediated macrophage immunosuppressive polarization via stabilizing IGF2BP3. Mol Cancer. 2022;21(1):16. 35031058 10.1186/s12943-021-01485-6 PMC8759291

[75] Ma R‐J , Ma C , Hu K , et al. Molecular mechanism, regulation, and therapeutic targeting of the STAT3 signaling pathway in esophageal cancer. Int J Oncol. 2022;61(3):1‐21. 10.3892/ijo.2022.5395 PMC9339493 35856449

[76] Ashrafizadeh M , Mohan CD , Rangappa S , et al. Noncoding RNAs as regulators of STAT3 pathway in gastrointestinal cancers: roles in cancer progression and therapeutic response. Med Res Rev. 2023;43(5):1263‐1321. 36951271 10.1002/med.21950

[77] Liu S , Li W , Liang L , et al. The regulatory relationship between transcription factor STAT3 and noncoding RNA. Cell Mol Biol Lett. 2024;29(1):4. 38172648 10.1186/s11658-023-00521-1 PMC10763091

[78] Li C‐J , Li D‐G , Liu E‐J , et al. Circ8199 encodes a protein that inhibits the activity of OGT by JAK2‐STAT3 pathway in esophageal squamous cell carcinoma. Am J Cancer Res. 2023;13(3):1107. 37034230 PMC10077033

[79] Hashemi M , Nadafzadeh N , Imani MH , et al. Targeting and regulation of autophagy in hepatocellular carcinoma: revisiting the molecular interactions and mechanisms for new therapy approaches. Cell Commun Signal. 2023;21(1):32. 36759819 10.1186/s12964-023-01053-z PMC9912665

[80] Song J , Liu Q , Han L , et al. Hsa_circ_0009092/miR‐665/NLK signaling axis suppresses colorectal cancer progression via recruiting TAMs in the tumor microenvironment. J Exp Clin Cancer Res. 2023;42(1):319. 38008713 10.1186/s13046-023-02887-8 PMC10680284

[81] Zheng X , Huang M , Xing L , et al. The circRNA circSEPT9 mediated by E2F1 and EIF4A3 facilitates the carcinogenesis and development of triple‐negative breast cancer. Mol Cancer. 2020;19:1‐22. 32264877 10.1186/s12943-020-01183-9 PMC7137343

[82] Wu S , Lu J , Zhu H , et al. A novel axis of circKIF4A‐miR‐637‐STAT3 promotes brain metastasis in triple‐negative breast cancer. Cancer Lett. 2024;581:216508. 38029538 10.1016/j.canlet.2023.216508

[83] Li Z , Ruan Y , Zhang H , et al. Tumor‐suppressive circular RNAs: mechanisms underlying their suppression of tumor occurrence and use as therapeutic targets. Cancer Sci. 2019;110(12):3630‐3638. 31599076 10.1111/cas.14211 PMC6890437

[84] Zhang Y , Li C , Liu X , et al. circHIPK3 promotes oxaliplatin‐resistance in colorectal cancer through autophagy by sponging miR‐637. EBioMedicine. 2019;48:277‐288. 31631038 10.1016/j.ebiom.2019.09.051 PMC6838436

[85] Yin H , Tang Q , Xia H , et al. Targeting RAF dimers in RAS mutant tumors: from biology to clinic. Acta Pharm Sin B. 2024;14(5):1895‐1923. 38799634 10.1016/j.apsb.2024.02.018 PMC11120325

[86] Klomp JE , Diehl JN , Klomp JA , et al. Determining the ERK‐regulated phosphoproteome driving KRAS‐mutant cancer. Science. 2024;384(6700):eadk0850. 38843329 10.1126/science.adk0850 PMC11301400

[87] Timofeev O , Giron P , Lawo S , et al. ERK pathway agonism for cancer therapy: evidence, insights, and a target discovery framework. NPJ Precis Oncol. 2024;8(1):70. 38485987 10.1038/s41698-024-00554-5 PMC10940698

[88] Meier C , La Rocca G , Nawrot V , et al. Erk inhibition as a promising therapeutic strategy for high IL‐8‐secreting and low SPTAN1‐expressing colorectal cancer. Int J Mol Sci. 2024;25(11):5658. 38891846 10.3390/ijms25115658 PMC11172072

[89] Klomp JA , Klomp JE , Stalnecker CA , et al. Defining the KRAS‐and ERK‐dependent transcriptome in KRAS‐mutant cancers. Science. 2024;384(6700):eadk0775. 38843331 10.1126/science.adk0775 PMC11301402

[90] Dong L , Choi H , Budhu S , et al. Intermittent MEK inhibition with GITR co‐stimulation rescues T‐cell function for increased efficacy with CTLA‐4 blockade in solid tumor models. Cancer Immunol Res. 2024;12(10):1392‐1408. 38885362 10.1158/2326-6066.CIR-23-0729 PMC12679059

[91] Lee S , Rauch J , Kolch W . Targeting MAPK signaling in cancer: mechanisms of drug resistance and sensitivity. Int J Mol Sci. 2020;21(3):1102. 32046099 10.3390/ijms21031102 PMC7037308

[92] Du J , Lan T , Liao H , et al. CircNFIB inhibits tumor growth and metastasis through suppressing MEK1/ERK signaling in intrahepatic cholangiocarcinoma. Mol Cancer. 2022;21(1):18. 35039066 10.1186/s12943-021-01482-9 PMC8762882

[93] Cui Y , Wu X , Jin J , et al. CircHERC1 promotes non‐small cell lung cancer cell progression by sequestering FOXO1 in the cytoplasm and regulating the miR‐142‐3p‐HMGB1 axis. Mol Cancer. 2023;22(1):179. 37932766 10.1186/s12943-023-01888-7 PMC10626661

[94] Shen Y , Zhang N , Chai J , et al. CircPDIA4 induces gastric cancer progression by promoting ERK1/2 activation and enhancing biogenesis of oncogenic circRNAs. Cancer Res. 2023;83(4):538‐552. 36562654 10.1158/0008-5472.CAN-22-1923

[95] Lyu Y , Tan B , Li L , et al. A novel protein encoded by circUBE4B promotes progression of esophageal squamous cell carcinoma by augmenting MAPK/ERK signaling. Cell Death Dis. 2023;14(6):346. 37264022 10.1038/s41419-023-05865-2 PMC10235080

[96] Zhang F , Wei D , Xie S , et al. CircZCCHC2 decreases pirarubicin sensitivity and promotes triple‐negative breast cancer development via the miR‐1200/TPR axis. Iscience. 2024;27(3). 10.1016/j.isci.2024.109057 PMC10867422 38361605

[97] Huang W , Hu X , He X , et al. TRIM29 facilitates gemcitabine resistance via MEK/ERK pathway and is modulated by circRPS29/miR‐770–5p axis in PDAC. Drug Resist Update. 2024;74:101079. 10.1016/j.drup.2024.101079 38518727

[98] Liu S , Wang Y , Wang T , et al. CircPCNXL2 promotes tumor growth and metastasis by interacting with STRAP to regulate ERK signaling in intrahepatic cholangiocarcinoma. Mol Cancer. 2024;23(1):35. 38365721 10.1186/s12943-024-01950-y PMC10873941

[99] Revathidevi S , Munirajan AK . Akt in cancer: mediator and more. Semin Cancer Biol. 2019;59:80‐91. 31173856 10.1016/j.semcancer.2019.06.002

[100] Pal I , Mandal M . PI3K and Akt as molecular targets for cancer therapy: current clinical outcomes. Acta Pharmacol Sin. 2012;33(12):1441‐1458. 22983389 10.1038/aps.2012.72 PMC4001841

[101] Li P , Huang D , Gu X . Exploring the dual role of circRNA and PI3K/AKT pathway in tumors of the digestive system. Biomed Pharmacother. 2023;168:115694. 37832407 10.1016/j.biopha.2023.115694

[102] Yan S , Chen L , Zhuang H , et al. HDAC inhibition sensitize hepatocellular carcinoma to lenvatinib via suppressing AKT activation. Int J Biol Sci. 2024;20(8):3046. 38904018 10.7150/ijbs.93375 PMC11186361

[103] Huang B , Ren J , Ma Q , et al. A novel peptide PDHK1‐241aa encoded by circPDHK1 promotes ccRCC progression via interacting with PPP1CA to inhibit AKT dephosphorylation and activate the AKT‐mTOR signaling pathway. Mol Cancer. 2024;23(1):34. 38360682 10.1186/s12943-024-01940-0 PMC10870583

[104] Zhao D , Dong Y , Duan M , et al. Circadian gene ARNTL initiates circGUCY1A2 transcription to suppress non‐small cell lung cancer progression via miR‐200c‐3p/PTEN signaling. J Exp Clin Cancer Res. 2023;42(1):229. 37667322 10.1186/s13046-023-02791-1 PMC10478228

[105] Ma Z , Chen H , Xia Z , et al. Energy stress‐induced circZFR enhances oxidative phosphorylation in lung adenocarcinoma via regulating alternative splicing. J Exp Clin Cancer Res. 2023;42(1):169. 37461053 10.1186/s13046-023-02723-z PMC10351155

[106] Liao W , Du J , Li L , et al. CircZNF215 promotes tumor growth and metastasis through inactivation of the PTEN/AKT pathway in intrahepatic cholangiocarcinoma. J Exp Clin Cancer Res. 2023;42(1):125. 37198696 10.1186/s13046-023-02699-w PMC10193609

[107] Zhang C , Yu Z , Yang S , et al. ZNF460‐mediated circRPPH1 promotes TNBC progression through ITGA5‐induced FAK/PI3K/AKT activation in a ceRNA manner. Mol Cancer. 2024;23(1):33. 38355583 10.1186/s12943-024-01944-w PMC10865535

[108] Li Y , Wang Z , Yang J , et al. CircTRIM1 encodes TRIM1‐269aa to promote chemoresistance and metastasis of TNBC via enhancing CaM‐dependent MARCKS translocation and PI3K/AKT/mTOR activation. Mol Cancer. 2024;23(1):102. 38755678 10.1186/s12943-024-02019-6 PMC11097450

[109] Lei K , Liang R , Liang J , et al. CircPDE5A‐encoded novel regulator of the PI3K/AKT pathway inhibits esophageal squamous cell carcinoma progression by promoting USP14‐mediated de‐ubiquitination of PIK3IP1. J Exp Clin Cancer Res. 2024;43(1):124. 38658954 10.1186/s13046-024-03054-3 PMC11040784

[110] Xue C , Li G , Zheng Q , et al. The functional roles of the circRNA/Wnt axis in cancer. Mol Cancer. 2022;21(1):108. 35513849 10.1186/s12943-022-01582-0 PMC9074313

[111] Li Y , Wang Z , Su P , et al. circ‐EIF6 encodes EIF6‐224aa to promote TNBC progression via stabilizing MYH9 and activating the Wnt/beta‐catenin pathway. Mol Ther. 2022;30(1):415‐430. 34450253 10.1016/j.ymthe.2021.08.026 PMC8753373

[112] Alimohammadi M , Gholinezhad Y , Mousavi V , et al. Circular RNAs: novel actors of Wnt signaling pathway in lung cancer progression. EXCLI J. 2023;22:645. 37636026 10.17179/excli2023-6209 PMC10450211

[113] Peng Y , Xu Y , Zhang X , et al. A novel protein AXIN1‐295aa encoded by circAXIN1 activates the Wnt/β‐catenin signaling pathway to promote gastric cancer progression. Mol Cancer. 2021;20:1‐19. 34863211 10.1186/s12943-021-01457-w PMC8642992

[114] Krishnamurthy N , Kurzrock R . Targeting the Wnt/beta‐catenin pathway in cancer: update on effectors and inhibitors. Cancer Treat Rev. 2018;62:50‐60. 29169144 10.1016/j.ctrv.2017.11.002 PMC5745276

[115] Li Y , Kong Y , An M , et al. ZEB1‐mediated biogenesis of circNIPBL sustains the metastasis of bladder cancer via Wnt/β‐catenin pathway. J Exp Clin Cancer Res. 2023;42(1):191. 37528489 10.1186/s13046-023-02757-3 PMC10394821

[116] Wu M , Qiu Q , Zhou Q , et al. circFBXO7/miR‐96‐5p/MTSS1 axis is an important regulator in the Wnt signaling pathway in ovarian cancer. Mol Cancer. 2022;21(1):137. 35768865 10.1186/s12943-022-01611-y PMC9241180

[117] Xu S , Luo C , Chen D , et al. circMMD reduction following tumor treating fields inhibits glioblastoma progression through FUBP1/FIR/DVL1 and miR‐15b‐5p/FZD6 signaling. J Exp Clin Cancer Res. 2023;42(1):64. 36932454 10.1186/s13046-023-02642-z PMC10021944

[118] Wang Z , Sun A , Yan A , et al. Circular RNA MTCL1 promotes advanced laryngeal squamous cell carcinoma progression by inhibiting C1QBP ubiquitin degradation and mediating beta‐catenin activation. Mol Cancer. 2022;21(1):92. 35366893 10.1186/s12943-022-01570-4 PMC8976408

[119] Zhu Q , Hu Y , Jiang W , et al. Circ‐CCT2 activates wnt/β‐catenin signaling to facilitate hepatoblastoma development by stabilizing PTBP1 mRNA. Cell Mol Gastroenterol. 2024;17(2):175‐197. 10.1016/j.jcmgh.2023.10.004 PMC10758885 37866478

[120] Kovall RA , Gebelein B , Sprinzak D , et al. The canonical Notch signaling pathway: structural and biochemical insights into shape, sugar, and force. Dev Cell. 2017;41(3):228‐241. 28486129 10.1016/j.devcel.2017.04.001 PMC5492985

[121] Meurette O , Mehlen P . Notch signaling in the tumor microenvironment. Cancer Cell. 2018;34(4):536‐548. 30146333 10.1016/j.ccell.2018.07.009

[122] Ferreira A , Aster JC . Notch signaling in cancer: complexity and challenges on the path to clinical translation. Semin Cancer Biol. 2022;85:95‐106. 33862222 10.1016/j.semcancer.2021.04.008

[123] Kunze B , Wein F , Fang H‐Y , et al. Notch signaling mediates differentiation in Barrett's esophagus and promotes progression to adenocarcinoma. Gastroenterology. 2020;159(2):575‐590. 32325086 10.1053/j.gastro.2020.04.033 PMC7484392

[124] Capaccione KM , Pine SR . The Notch signaling pathway as a mediator of tumor survival. Carcinogenesis. 2013;34(7):1420‐1430. 23585460 10.1093/carcin/bgt127 PMC3697894

[125] Ai J , Zhang W , Deng W , et al. A hsa_circ_001726 axis regulated by E2F6 contributes to metastasis of hepatocellular carcinoma. BMC Cancer. 2024;24(1):14. 38166853 10.1186/s12885-023-11703-7 PMC10763683

[126] Chen L , Yang X , Zhao J , et al. Circ_0008532 promotes bladder cancer progression by regulation of the miR‐155‐5p/miR‐330‐5p/MTGR1 axis. J Exp Clin Cancer Res. 2020;39:1‐12. 32460831 10.1186/s13046-020-01592-0 PMC7251916

[127] Buratin A , Borin C , et al. CircFBXW7 in patients with T‐cell ALL: depletion sustains MYC and NOTCH activation and leukemia cell viability. Exp Hematol Oncol. 2023;12(1):12. 36681829 10.1186/s40164-023-00374-6 PMC9863195

[128] Chen J , Hei R , Chen C , et al. CircCRIM1 suppresses osteosarcoma progression via sponging miR146a‐5p and targeting NUMB. Am J Cancer Res. 2023;13(8):3463. 37693139 PMC10492126

[129] Long F , Lin Z , Li L , et al. Comprehensive landscape and future perspectives of circular RNAs in colorectal cancer. Mol Cancer. 2021;20(1):26. 33536039 10.1186/s12943-021-01318-6 PMC7856739

[130] Wang S , Dong Y , Gong A , et al. Exosomal circRNAs as novel cancer biomarkers: challenges and opportunities. Int J Biol Sci. 2021;17(2):562. 33613113 10.7150/ijbs.48782 PMC7893596

[131] Xie G , Lei B , Yin Z , et al. Circ MTA2 drives gastric cancer progression through suppressing MTA2 degradation via interacting with UCHL3. Int J Mol Sci. 2024;25(5):2817. 38474064 10.3390/ijms25052817 PMC10932366

[132] Wang H , Tang Z , Duan J , et al. Cancer‐released exosomal circular RNA circ_0008717 promotes cell tumorigenicity through microRNA‐1287‐5p/P21‐activated kinase 2 (PAK2) axis in non‐small cell lung cancer. Bioengineered. 2022;13(4):8937‐8949. 35333693 10.1080/21655979.2022.2056822 PMC9161925

[133] Wang Y , Zou R , Li D , et al. Exosomal circSTRBP from cancer cells facilitates gastric cancer progression via regulating miR‐1294/miR‐593‐3p/E2F2 axis. J Cell Mol Med. 2024;28(8):e18217. 38520208 10.1111/jcmm.18217 PMC10960172

[134] Li B , Chen J , Wu Y , et al. Decrease of circARID1A retards glioblastoma invasion by modulating miR‐370‐3p/TGFBR2 pathway. Int J Biol Sci. 2022;18(13):5123. 35982888 10.7150/ijbs.66673 PMC9379412

[135] Luo A , Liu H , Huang C , et al. Exosome‐transmitted circular RNA circ‐LMO7 facilitates the progression of osteosarcoma by regulating miR‐21‐5p/ARHGAP24 axis. Cancer Biol Ther. 2024;25(1):2343450. 38742566 10.1080/15384047.2024.2343450 PMC11095575

[136] Zhang C , Wei G , Zhu X , et al. Exosome‐delivered circSTAU2 inhibits the progression of gastric cancer by targeting the miR‐589/CAPZA1 Axis. Int J Nanomed. 2023:127‐142. 10.2147/IJN.S391872 PMC9832994 36643863

[137] Luo Y , Zhu Q , Xiang S , et al. Downregulated circPOKE promotes breast cancer metastasis through activation of the USP10‐Snail axis. Oncogene. 2023;42(44):3236‐3251. 37717099 10.1038/s41388-023-02823-2

[138] Chen C , Liu Y , Liu L , et al. Exosomal circTUBGCP4 promotes vascular endothelial cell tipping and colorectal cancer metastasis by activating Akt signaling pathway. J Exp Clin Cancer Res. 2023;42(1):46. 36793126 10.1186/s13046-023-02619-y PMC9930311

[139] Hong W , Du K , Zhang Q , et al. Tanreqing suppresses the proliferation and migration of non‐small cell lung cancer cells by mediating the inactivation of the HIF1α signaling pathway via exosomal circ‐WDR78. J Biomol Struct Dyn. 2024:1‐12. 10.1080/07391102.2023.2301514 38247231

[140] Ghafouri I , Pakravan K , Razmara E , et al. Colorectal cancer‐secreted exosomal circ_001422 plays a role in regulating KDR expression and activating mTOR signaling in endothelial cells by targeting miR‐195‐5p. J Cancer Res Clin. 2023;149(13):12227‐12240. 10.1007/s00432-023-05095-1 PMC11797106 37432457

[141] Mao Y , Wang J , Wang Y , et al. Hypoxia induced exosomal Circ‐ZNF609 promotes pre‐metastatic niche formation and cancer progression via miR‐150‐5p/VEGFA and HuR/ZO‐1 axes in esophageal squamous cell carcinoma. Cell Death Discov. 2024;10(1):133. 38472174 10.1038/s41420-024-01905-8 PMC10933275

[142] Miao Z , Zhao X , Liu X . Exosomal circCOL1A2 from cancer cells accelerates colorectal cancer progression via regulating miR‐665/LASP1 signal axis. Eur J Pharmacol. 2023;950:175722. 37059374 10.1016/j.ejphar.2023.175722

[143] Jiang W , Yu Y , Ou J , et al. Exosomal circRNA RHOT1 promotes breast cancer progression by targeting miR‐204‐5p/PRMT5 axis. Cancer Cell Int. 2023;23(1):260. 37924099 10.1186/s12935-023-03111-5 PMC10623849

[144] Liu L , Liao R , Wu Z , et al. Hepatic stellate cell exosome‐derived circWDR25 promotes the progression of hepatocellular carcinoma via the miRNA‐4474‐3P‐ALOX‐15 and EMT axes. BioSci Trends. 2022;16(4):267‐281. 35934785 10.5582/bst.2022.01281

[145] Sang H , Zhang W , Peng L , et al. Exosomal circRELL1 serves as a miR‐637 sponge to modulate gastric cancer progression via regulating autophagy activation. Cell Death Dis. 2022;13(1):56. 35027539 10.1038/s41419-021-04364-6 PMC8758736

[146] Wang X , Dong F‐L , Wang Y‐Q , et al. Exosomal circTGFBR2 promotes hepatocellular carcinoma progression via enhancing ATG5 mediated protective autophagy. Cell Death Dis. 2023;14(7):451. 37474520 10.1038/s41419-023-05989-5 PMC10359294

[147] Song H , Zhao Z , Ma L , et al. Novel exosomal circEGFR facilitates triple negative breast cancer autophagy via promoting TFEB nuclear trafficking and modulating miR‐224‐5p/ATG13/ULK1 feedback loop. Oncogene. 2024:1‐16. 38280941 10.1038/s41388-024-02950-4 PMC10920198

[148] Liu Y , Ma L , Hua F , et al. Exosomal circCARM1 from spheroids reprograms cell metabolism by regulating PFKFB2 in breast cancer. Oncogene. 2022;41(14):2012‐2025. 35027669 10.1038/s41388-021-02061-4

[149] Lin J , Wang X , Zhai S , et al. Hypoxia‐induced exosomal circPDK1 promotes pancreatic cancer glycolysis via c‐myc activation by modulating miR‐628‐3p/BPTF axis and degrading BIN1. J Hematol Oncol. 2022;15(1):128. 36068586 10.1186/s13045-022-01348-7 PMC9450374

[150] Huang C , Zhou Y , Feng X , et al. Delivery of engineered primary tumor‐derived exosomes effectively suppressed the colorectal cancer chemoresistance and liver metastasis. ACS Nano. 2023;17(11):10313‐10326. 37141393 10.1021/acsnano.3c00668

[151] Chen Y , Liu H , Zou J , et al. Exosomal circ_0091741 promotes gastric cancer cell autophagy and chemoresistance via the miR‐330‐3p/TRIM14/Dvl2/Wnt/β‐catenin axis. Hum Cell. 2023;36(1):258‐275. 36323918 10.1007/s13577-022-00790-6

[152] Zhang Y , Tan X , Lu Y . Exosomal transfer of circ_0006174 contributes to the chemoresistance of doxorubicin in colorectal cancer by depending on the miR‐1205/CCND2 axis. J Physiol Biochem. 2022;78(1):39‐50. 34792792 10.1007/s13105-021-00831-y

[153] Culy CR , Clemett D , Wiseman LR . Oxaliplatin: a review of its pharmacological properties and clinical efficacy in metastatic colorectal cancer and its potential in other malignancies. Drugs. 2000;60:895‐924. 11085200 10.2165/00003495-200060040-00005

[154] Pan Z , Zheng J , Zhang J , et al. A novel protein encoded by exosomal CircATG4B induces oxaliplatin resistance in colorectal cancer by promoting autophagy. Adv Sci. 2022;9(35):2204513. 10.1002/advs.202204513 PMC9762280 36285810

[155] Wu Y , Xu M , Feng Z , et al. AUF1‐induced circular RNA hsa_circ_0010467 promotes platinum resistance of ovarian cancer through miR‐637/LIF/STAT3 axis. Cell Mol Life Sci. 2023;80(9):256. 37589744 10.1007/s00018-023-04906-5 PMC11072515

[156] Chen Z , Xu Z , Wang Q , et al. Exosome‐delivered circRNA circSYT15 contributes to cisplatin resistance in cervical cancer cells through the miR‐503‐5p/RSF1 axis. Cell Cycle. 2023;22(20):2211‐2228. 37974391 10.1080/15384101.2023.2281768 PMC10730224

[157] Gong J , Han G , Chen Z , et al. CircDCAF8 promotes the progression of hepatocellular carcinoma through miR‐217/NAP1L1 Axis, and induces angiogenesis and regorafenib resistance via exosome‐mediated transfer. J Transl Med. 2024;22(1):1‐20. 38816735 10.1186/s12967-024-05233-4 PMC11137954

[158] Dong F‐L , Xu Z‐Z , Wang Y‐Q , et al. Exosome‐derived circUPF2 enhances resistance to targeted therapy by redeploying ferroptosis sensitivity in hepatocellular carcinoma. J Nanobiotechnol. 2024;22(1):298. 10.1186/s12951-024-02582-6 PMC11137910 38811968

[159] Xia W , Chen W , Ni C , et al. Chemotherapy‐induced exosomal circBACH1 promotes breast cancer resistance and stemness via miR‐217/G3BP2 signaling pathway. Breast Cancer Res. 2023;25(1):85. 37461019 10.1186/s13058-023-01672-x PMC10351125

[160] Wang X , Chen T , Li C , et al. CircRNA‐CREIT inhibits stress granule assembly and overcomes doxorubicin resistance in TNBC by destabilizing PKR. J Hematol Oncol. 2022;15(1):122. 36038948 10.1186/s13045-022-01345-w PMC9425971

[161] Wei S‐L , Ye J‐J , Sun L , et al. Exosome‐derived circKIF20B suppresses gefitinib resistance and cell proliferation in non‐small cell lung cancer. Cancer Cell Int. 2023;23(1):129. 37394466 10.1186/s12935-023-02974-y PMC10316567

[162] Lu L , Zeng Y , Yu Z , et al. EIF4a3‐regulated circRABL2B regulates cell stemness and drug sensitivity of lung cancer via YBX1‐dependent downregulation of MUC5AC expression. Int J Biol Sci. 2023;19(9):2725. 37324942 10.7150/ijbs.78588 PMC10266078

[163] Luo X , Li Y , Hua Z , et al. Exosomes‐mediated tumor metastasis through reshaping tumor microenvironment and distant niche. J Control Release. 2023;353:327‐336. 36464063 10.1016/j.jconrel.2022.11.050

[164] Elhanani O , Ben‐Uri R , Keren L . Spatial profiling technologies illuminate the tumor microenvironment. Cancer Cell. 2023;41(3):404‐420. 36800999 10.1016/j.ccell.2023.01.010

[165] Toninelli M , Rossetti G , Pagani M . Charting the tumor microenvironment with spatial profiling technologies. Trends Cancer. 2023;9(12):1085‐1096. 37673713 10.1016/j.trecan.2023.08.004

[166] Marangon D , Lecca D . Exosomal non‐coding RNAs in glioma progression: insights into tumor microenvironment dynamics and therapeutic implications. Front Cell Dev Biol. 2023;11:1275755. 38020906 10.3389/fcell.2023.1275755 PMC10646304

[167] Tsoumakidou M . The advent of immune stimulating CAFs in cancer. Nat Rev Cancer. 2023;23(4):258‐269. 36807417 10.1038/s41568-023-00549-7

[168] Kim S‐J , Khadka D , Seo JH . Interplay between solid tumors and tumor microenvironment. Front Immunol. 2022;13:882718. 35707536 10.3389/fimmu.2022.882718 PMC9189309

[169] Li Y , Zheng X , Wang J , et al. Exosomal circ‐AHCY promotes glioblastoma cell growth via Wnt/β‐catenin signaling pathway. Ann Clin Transl Neur. 2023;10(6):865‐878. 10.1002/acn3.51743 PMC10270256 37150844

[170] Dai W , Wu X , Li J , et al. Hedgehog‐Gli1‐derived exosomal circ‐0011536 mediates peripheral neural remodeling in pancreatic cancer by modulating the miR‐451a/VGF axis. J Exp Clin Cancer Res. 2023;42(1):329. 38041128 10.1186/s13046-023-02894-9 PMC10693175

[171] Ding L , Zheng Q , Lin Y , et al. Exosome‐derived circTFDP2 promotes prostate cancer progression by preventing PARP1 from caspase‐3‐dependent cleavage. Clin Transl Med. 2023;13(1):e1156. 36597139 10.1002/ctm2.1156 PMC9810792

[172] Chen L , Guo P , He Y , et al. HCC‐derived exosomes elicit HCC progression and recurrence by epithelial‐mesenchymal transition through MAPK/ERK signalling pathway. Cell Death Dis. 2018;9(5):513. 29725020 10.1038/s41419-018-0534-9 PMC5938707

[173] Huang X‐Y , Huang Z‐L , Huang J , et al. Exosomal circRNA‐100338 promotes hepatocellular carcinoma metastasis via enhancing invasiveness and angiogenesis. J Exp Clin Cancer Res. 2020;39:1‐16. 31973767 10.1186/s13046-020-1529-9 PMC6979009

[174] Zhang H , Yue X , Chen Z , et al. Define cancer‐associated fibroblasts (CAFs) in the tumor microenvironment: new opportunities in cancer immunotherapy and advances in clinical trials. Mol Cancer. 2023;22(1):159. 37784082 10.1186/s12943-023-01860-5 PMC10544417

[175] Öhlund D , Handly‐Santana A , Biffi G , et al. Distinct populations of inflammatory fibroblasts and myofibroblasts in pancreatic cancer. J Exp Med. 2017;214(3):579‐596. 28232471 10.1084/jem.20162024 PMC5339682

[176] Fang Z , Meng Q , Xu J , et al. Signaling pathways in cancer‐associated fibroblasts: recent advances and future perspectives. Cancer Commun. 2023;43(1):3‐41. 10.1002/cac2.12392 PMC9859735 36424360

[177] Kennel KB , Bozlar M , De Valk AF , et al. Cancer‐associated fibroblasts in inflammation and antitumor immunity. Clin Cancer Res. 2023;29(6):1009‐1016. 36399325 10.1158/1078-0432.CCR-22-1031 PMC10011884

[178] Ye F , Liang Y , Wang Y , et al. Cancer‐associated fibroblasts facilitate breast cancer progression through exosomal circTBPL1‐mediated intercellular communication. Cell Death Dis. 2023;14(7):471. 37495592 10.1038/s41419-023-05986-8 PMC10372047

[179] Jiang Q , Lei Z , Wang Z , et al. Tumor‐associated fibroblast‐derived exosomal circDennd1b promotes pituitary adenoma progression by modulating the miR‐145‐5p/ONECUT2 axis and activating the MAPK pathway. Cancers. 2023;15(13):3375. 37444485 10.3390/cancers15133375 PMC10340501

[180] Yang C , Zhang Y , Yan M , et al. Exosomes derived from cancer‐associated fibroblasts promote tumorigenesis, metastasis and chemoresistance of colorectal cancer by upregulating circ_0067557 to target Lin28. BMC Cancer. 2024;24(1):64. 38216964 10.1186/s12885-023-11791-5 PMC10785442

[181] Nilsson I , Bahram F , Li X , et al. VEGF receptor 2/‐3 heterodimers detected in situ by proximity ligation on angiogenic sprouts. EMBO J. 2010;29(8):1377‐1388. 20224550 10.1038/emboj.2010.30 PMC2868571

[182] Bautch VL . Endothelial cells form a phalanx to block tumor metastasis. Cell. 2009;136(5):810‐812. 19269358 10.1016/j.cell.2009.02.021

[183] Coso S , Bovay E , Petrova TV . Pressing the right buttons: signaling in lymphangiogenesis. Blood. 2014;123(17):2614‐2624. 24608974 10.1182/blood-2013-12-297317

[184] Sheldon H , Heikamp E , Turley H , et al. New mechanism for Notch signaling to endothelium at a distance by Delta‐like 4 incorporation into exosomes. Blood. 2010;116(13):2385‐2394. 20558614 10.1182/blood-2009-08-239228

[185] Bovy N , Blomme B , Frères P , et al. Endothelial exosomes contribute to the antitumor response during breast cancer neoadjuvant chemotherapy via microRNA transfer. Oncotarget. 2015;6(12):10253. 25860935 10.18632/oncotarget.3520 PMC4496353

[186] Maes H , Olmeda D , Soengas MS , et al. Vesicular trafficking mechanisms in endothelial cells as modulators of the tumor vasculature and targets of antiangiogenic therapies. FEBS J. 2016;283(1):25‐38. 26443003 10.1111/febs.13545

[187] Shen X , Kong S , Ma S , et al. Hsa_circ_0000437 promotes pathogenesis of gastric cancer and lymph node metastasis. Oncogene. 2022;41(42):4724‐4735. 36109630 10.1038/s41388-022-02449-w

[188] Thommen DS , Schumacher TN . T cell dysfunction in cancer. Cancer Cell. 2018;33(4):547‐562. 29634943 10.1016/j.ccell.2018.03.012 PMC7116508

[189] Reina‐Campos M , Scharping NE , Goldrath AW . CD8^+^ T cell metabolism in infection and cancer. Nat Rev Immunol. 2021;21(11):718‐738. 33981085 10.1038/s41577-021-00537-8 PMC8806153

[190] Hashimoto M , Kamphorst AO , Im SJ , et al. CD8 T cell exhaustion in chronic infection and cancer: opportunities for interventions. Annu Rev Med. 2018;69:301‐318. 29414259 10.1146/annurev-med-012017-043208

[191] Park J , Hsueh P‐C , Li Z , et al. Microenvironment‐driven metabolic adaptations guiding CD8^+^ T cell anti‐tumor immunity. Immunity. 2023;56(1):32‐42. 36630916 10.1016/j.immuni.2022.12.008

[192] Dolina JS , Van Braeckel‐Budimir N , Thomas GD , et al. CD8^+^ T cell exhaustion in cancer. Front Immunol. 2021;12:715234. 34354714 10.3389/fimmu.2021.715234 PMC8330547

[193] Wang J , Zhao X , Wang Y , et al. circRNA‐002178 act as a ceRNA to promote PDL1/PD1 expression in lung adenocarcinoma. Cell Death Dis. 2020;11(1):32. 31949130 10.1038/s41419-020-2230-9 PMC6965119

[194] Chen S‐W , Zhu S‐Q , Pei X , et al. Cancer cell‐derived exosomal circUSP7 induces CD8^+^ T cell dysfunction and anti‐PD1 resistance by regulating the miR‐934/SHP2 axis in NSCLC. Mol Cancer. 2021;20:1‐18. 34753486 10.1186/s12943-021-01448-x PMC8576933

[195] Luo Y‐H , Yang Y‐P , Chien C‐S , et al. Circular RNA hsa_circ_0000190 facilitates the tumorigenesis and immune evasion by upregulating the expression of soluble PD‐L1 in non‐small‐cell lung cancer. Int J Mol Sci. 2021;23(1):64. 35008490 10.3390/ijms23010064 PMC8744551

[196] Hu Z , Chen G , Zhao Y , et al. Exosome‐derived circCCAR1 promotes CD8^+^ T‐cell dysfunction and anti‐PD1 resistance in hepatocellular carcinoma. Mol Cancer. 2023;22(1):55. 36932387 10.1186/s12943-023-01759-1 PMC10024440

[197] Lu J‐C , Zhang P‐F , Huang X‐Y , et al. Amplification of spatially isolated adenosine pathway by tumor–macrophage interaction induces anti‐PD1 resistance in hepatocellular carcinoma. J Hematol Oncol. 2021;14:1‐20. 34838121 10.1186/s13045-021-01207-x PMC8627086

[198] Yang C , Wu S , Mou Z , et al. Exosome‐derived circTRPS1 promotes malignant phenotype and CD8^+^ T cell exhaustion in bladder cancer microenvironments. Mol Ther. 2022;30(3):1054‐1070. 35038580 10.1016/j.ymthe.2022.01.022 PMC8899700

[199] Shan F , Somasundaram A , Bruno TC , et al. Therapeutic targeting of regulatory T cells in cancer. Trends Cancer. 2022;8(11):944‐961. 35853825 10.1016/j.trecan.2022.06.008 PMC9588644

[200] Li C , Jiang P , Wei S , et al. Regulatory T cells in tumor microenvironment: new mechanisms, potential therapeutic strategies and future prospects. Mol Cancer. 2020;19:1‐23. 32680511 10.1186/s12943-020-01234-1 PMC7367382

[201] Alvisi G , Termanini A , Soldani C , et al. Multimodal single‐cell profiling of intrahepatic cholangiocarcinoma defines hyperactivated Tregs as a potential therapeutic target. J Hepatol. 2022;77(5):1359‐1372. 35738508 10.1016/j.jhep.2022.05.043

[202] Huang M , Huang X , Huang N . Exosomal circGSE1 promotes immune escape of hepatocellular carcinoma by inducing the expansion of regulatory T cells. Cancer Sci. 2022;113(6):1968‐1983. 35396771 10.1111/cas.15365 PMC9207376

[203] Chen Y , Li Z , Liang J , et al. CircRNA has_circ_0069313 induced OSCC immunity escape by miR‐325‐3p‐Foxp3 axes in both OSCC cells and Treg cells. Aging (Albany NY). 2022;14(10):4376. 35575762 10.18632/aging.204068 PMC9186771

[204] Li C , Xu X , Wei S , et al. Tumor‐associated macrophages: potential therapeutic strategies and future prospects in cancer. J Immunother Cancer. 2021;9(1). 10.1136/jitc-2020-001341 PMC8728363 33504575

[205] Vinogradov S , Warren G , Wei X . Macrophages associated with tumors as potential targets and therapeutic intermediates. Nanomedicine. 2014;9(5):695‐707. 24827844 10.2217/nnm.14.13 PMC4149280

[206] Xu M , Zhou C , Weng J , et al. Tumor associated macrophages‐derived exosomes facilitate hepatocellular carcinoma malignance by transferring lncMMPA to tumor cells and activating glycolysis pathway. J Exp Clin Cancer Res. 2022;41(1):253. 35986343 10.1186/s13046-022-02458-3 PMC9389814

[207] Lu Y , Han G , Zhang Y , et al. M2 macrophage‐secreted exosomes promote metastasis and increase vascular permeability in hepatocellular carcinoma. Cell Commun Signal. 2023;21(1):299. 37904170 10.1186/s12964-022-00872-w PMC10614338

[208] Ran X‐M , Yang J , Wang Z‐Y , et al. M2 macrophage‐derived exosomal circTMCO3 acts through miR‐515‐5p and ITGA8 to enhance malignancy in ovarian cancer. Commun Biol. 2024;7(1):583. 38755265 10.1038/s42003-024-06095-8 PMC11098810

[209] Wang L , Yi X , Xiao X , et al. Exosomal miR‐628‐5p from M1 polarized macrophages hinders m6A modification of circFUT8 to suppress hepatocellular carcinoma progression. Cell Mol Biol Lett. 2022;27(1):106. 36474147 10.1186/s11658-022-00406-9 PMC9724320

[210] Chen M , Cao C , Ma J . Tumor‐related exosomal circ_0001715 promotes lung adenocarcinoma cell proliferation and metastasis via enhancing M2 macrophage polarization by regulating triggering receptor expressed on myeloid cells‐2. Thorac Cancer. 2024;15(3):227‐238. 38087801 10.1111/1759-7714.15182 PMC10803224

[211] Zhang L , Zhang J , Li P , et al. Exosomal hsa_circ_0004658 derived from RBPJ overexpressed‐macrophages inhibits hepatocellular carcinoma progression via miR‐499b‐5p/JAM3. Cell Death Dis. 2022;13(1):32. 35013102 10.1038/s41419-021-04345-9 PMC8748962

[212] Shi L , Cao Y , Yuan W , et al. Exosomal circRNA BTG2 derived from RBP‐J overexpressed‐macrophages inhibits glioma progression via miR‐25‐3p/PTEN. Cell Death Dis. 2022;13(5):506. 35643814 10.1038/s41419-022-04908-4 PMC9148311

[213] Deng C , Huo M , Chu H , et al. Exosome circATP8A1 induces macrophage M2 polarization by regulating the miR‐1‐3p/STAT6 axis to promote gastric cancer progression. Mol Cancer. 2024;23(1):49. 38459596 10.1186/s12943-024-01966-4 PMC10921793

[214] Zhang Y , Guo J , Zhang L , et al. CircASPH enhances exosomal STING to facilitate M2 macrophage polarization in colorectal cancer. Inflamm Bowel Dis. 2023;29(12):1941‐1956. 37624989 10.1093/ibd/izad113

[215] Zhou B , Mo Z , Lai G , et al. Targeting tumor exosomal circular RNA cSERPINE2 suppresses breast cancer progression by modulating MALT1‐NF‐𝜅B‐IL‐6 axis of tumor‐associated macrophages. J Exp Clin Canc Res. 2023;42(1):48. 10.1186/s13046-023-02620-5 PMC9936722 36797769

[216] Sun Z , Xu Y , Shao B , et al. Exosomal circPOLQ promotes macrophage M2 polarization via activating IL‐10/STAT3 axis in a colorectal cancer model. J Immunother Cancer. 2024;12(5). 10.1136/jitc-2023-008491 PMC11116870 38782541

[217] Zhang Y , Wang X , Liu W , et al. CircGLIS3 promotes gastric cancer progression by regulating the miR‐1343‐3p/PGK1 pathway and inhibiting vimentin phosphorylation. J Transl Med. 2024;22(1):251. 38459513 10.1186/s12967-023-04625-2 PMC10921581

[218] Lu C , Shi W , Hu W , et al. Endoplasmic reticulum stress promotes breast cancer cells to release exosomes circ_0001142 and induces M2 polarization of macrophages to regulate tumor progression. Pharmacol Res. 2022;177:106098. 35091089 10.1016/j.phrs.2022.106098

[219] Wang D , Wang S , Jin M , et al. Hypoxic exosomal circPLEKHM1‐mediated crosstalk between tumor cells and macrophages drives lung cancer metastasis. Adv Sci. 2024:2309857. 10.1002/advs.202309857 PMC11165461 38509870

[220] Katopodi T , Petanidis S , Domvri K , et al. Kras‐driven intratumoral heterogeneity triggers infiltration of M2 polarized macrophages via the circHIPK3/PTK2 immunosuppressive circuit. Sci Rep. 2021;11(1):15455. 34326381 10.1038/s41598-021-94671-x PMC8322174

[221] Myers JA , Miller JS . Exploring the NK cell platform for cancer immunotherapy. Nat Rev Clin Oncol. 2021;18(2):85‐100. 32934330 10.1038/s41571-020-0426-7 PMC8316981

[222] Wu S‐Y , Fu T , Jiang Y‐Z , et al. Natural killer cells in cancer biology and therapy. Mol Cancer. 2020;19:1‐26. 32762681 10.1186/s12943-020-01238-x PMC7409673

[223] Lamers‐Kok N , Panella D , Georgoudaki A‐M , et al. Natural killer cells in clinical development as non‐engineered, engineered, and combination therapies. J Hematol Oncol. 2022;15(1):164. 36348457 10.1186/s13045-022-01382-5 PMC9644572

[224] Vivier E , Rebuffet L , Narni‐Mancinelli E , et al. Natural killer cell therapies. Nature. 2024;626(8000):727‐736. 38383621 10.1038/s41586-023-06945-1

[225] Bald T , Krummel MF , Smyth MJ , et al. The NK cell‐cancer cycle: advances and new challenges in NK cell‐based immunotherapies. Nat Immunol. 2020;21(8):835‐847. 32690952 10.1038/s41590-020-0728-z PMC8406687

[226] Zhang P‐F , Gao C , Huang X‐Y , et al. Cancer cell‐derived exosomal circUHRF1 induces natural killer cell exhaustion and may cause resistance to anti‐PD1 therapy in hepatocellular carcinoma. Mol Cancer. 2020;19:1‐15. 32593303 10.1186/s12943-020-01222-5 PMC7320583

[227] Lasser SA , Arkhypov I , et al. Myeloid‐derived suppressor cells in cancer and cancer therapy. Nat Rev Clin Oncol. 2024;21(2):147‐164. 38191922 10.1038/s41571-023-00846-y

[228] Hegde S , Leader AM , Merad M . MDSC: markers, development, states, and unaddressed complexity. Immunity. 2021;54(5):875‐884. 33979585 10.1016/j.immuni.2021.04.004 PMC8709560

[229] Gao F , Xu Q , Tang Z , et al. Exosomes derived from myeloid‐derived suppressor cells facilitate castration‐resistant prostate cancer progression via S100A9/circMID1/miR‐506‐3p/MID1. J Transl Med. 2022;20(1):346. 35918733 10.1186/s12967-022-03494-5 PMC9344715

[230] Liu X , Zhao S , Sui H , et al. MicroRNAs/LncRNAs modulate MDSCs in tumor microenvironment. Front Oncol. 2022;12:772351. 35359390 10.3389/fonc.2022.772351 PMC8963964

[231] Zhou X , Fang D , Liu H , et al. PMN‐MDSCs accumulation induced by CXCL1 promotes CD8^+^ T cells exhaustion in gastric cancer. Cancer Lett. 2022;532:215598. 35176418 10.1016/j.canlet.2022.215598

[232] Pettinella F , Mariotti B , Lattanzi C , et al. Surface CD52, CD84, and PTGER2 mark mature PMN‐MDSCs from cancer patients and G‐CSF‐treated donors. Cell Rep Med. 2024;5(2). 10.1016/j.xcrm.2023.101380 PMC10897522 38242120

[233] Zhang R , Dong M , Tu J , et al. PMN‐MDSCs modulated by CCL20 from cancer cells promoted breast cancer cell stemness through CXCL2‐CXCR2 pathway. Signal Transduct Target Ther. 2023;8(1):97. 36859354 10.1038/s41392-023-01337-3 PMC9977784

[234] Shi X , Pang S , Zhou J , et al. Bladder‐cancer‐derived exosomal circRNA_0013936 promotes suppressive immunity by up‐regulating fatty acid transporter protein 2 and down‐regulating receptor‐interacting protein kinase 3 in PMN‐MDSCs. Mol Cancer. 2024;23(1):52. 38461272 10.1186/s12943-024-01968-2 PMC10924381

[235] Xiong S , Dong L , Cheng L . Neutrophils in cancer carcinogenesis and metastasis. J Hematol Oncol. 2021;14:1‐17. 34674757 10.1186/s13045-021-01187-y PMC8529570

[236] Jaillon S , Ponzetta A , Di Mitri D , et al. Neutrophil diversity and plasticity in tumour progression and therapy. Nat Rev Cancer. 2020;20(9):485‐503. 32694624 10.1038/s41568-020-0281-y

[237] Aroca‐Crevillén A , Vicanolo T , Ovadia S , et al. Neutrophils in physiology and pathology. Annu Rev Pathol‐Mech. 2024;19(1):227‐259. 10.1146/annurev-pathmechdis-051222-015009 PMC11060889 38265879

[238] Que H , Fu Q , Lan T , et al. Tumor‐associated neutrophils and neutrophil‐targeted cancer therapies. BBA‐Rev Cancer. 2022;1877(5):188762. 10.1016/j.bbcan.2022.188762 35853517

[239] Liu S , Wu W , Du Y , et al. The evolution and heterogeneity of neutrophils in cancers: origins, subsets, functions, orchestrations and clinical applications. Mol Cancer. 2023;22(1):148. 37679744 10.1186/s12943-023-01843-6 PMC10483725

[240] Shaul ME , Fridlender ZG . Tumour‐associated neutrophils in patients with cancer. Nat Rev Clin Oncol. 2019;16(10):601‐620. 31160735 10.1038/s41571-019-0222-4

[241] Shang A , Gu C , Wang W , et al. Exosomal circPACRGL promotes progression of colorectal cancer via the miR‐142‐3p/miR‐506‐3p‐TGF‐β1 axis. Mol Cancer. 2020;19:1‐15. 32713345 10.1186/s12943-020-01235-0 PMC7384220

[242] Park EM , Chelvanambi M , Bhutiani N , et al. Targeting the gut and tumor microbiota in cancer. Nat Med. 2022;28(4):690‐703. 35440726 10.1038/s41591-022-01779-2

[243] Yang L , Li A , Wang Y , et al. Intratumoral microbiota: roles in cancer initiation, development and therapeutic efficacy. Signal Transduct Target Ther. 2023;8(1):35. 36646684 10.1038/s41392-022-01304-4 PMC9842669

[244] Sepich‐Poore GD , Zitvogel L , Straussman R , et al. The microbiome and human cancer. Science. 2021;371(6536):eabc4552. 33766858 10.1126/science.abc4552 PMC8767999

[245] Garrett WS . Cancer and the microbiota. Science. 2015;348(6230):80‐86. 25838377 10.1126/science.aaa4972 PMC5535753

[246] Zang X , Wang R , Wang Z , et al. Exosomal circ50547 as a potential marker and promotor of gastric cancer progression via miR‐217/HNF1B axis. Transl Oncol. 2024;45:101969. 38692196 10.1016/j.tranon.2024.101969 PMC11070923

[247] Huang X‐j , Wang Y , Wang H‐t , et al. Exosomal hsa_circ_000200 as a potential biomarker and metastasis enhancer of gastric cancer via miR‐4659a/b‐3p/HBEGF axis. Cancer Cell Int. 2023;23(1):151. 37525152 10.1186/s12935-023-02976-w PMC10391853

[248] Chen Z , Ma X , Chen Z , et al. Exosome‐transported circ_0061407 and circ_0008103 play a tumour‐repressive role and show diagnostic value in non‐small‐cell lung cancer. J Transl Med. 2024;22(1):427. 38711144 10.1186/s12967-024-05215-6 PMC11071259

[249] Li X , Lin Y‐L , Shao J‐K , et al. Plasma exosomal hsa_circ_0079439 as a novel biomarker for early detection of gastric cancer. World J Gastroenterol. 2023;29(22):3482. 37389236 10.3748/wjg.v29.i22.3482 PMC10303519

[250] Liu Q , Li S . Exosomal circRNAs: novel biomarkers and therapeutic targets for urinary tumors. Cancer Lett. 2024:216759. 38417667 10.1016/j.canlet.2024.216759

[251] Gopikrishnan M , Ashour HM , Pintus G , et al. Therapeutic and diagnostic applications of exosomal circRNAs in breast cancer. Funct Integr Genomic. 2023;23(2):184. 10.1007/s10142-023-01083-3 PMC10224846 37243750

[252] Zheng R , Zhang K , Tan S , et al. Exosomal circLPAR1 functions in colorectal cancer diagnosis and tumorigenesis through suppressing BRD4 via METTL3‐eIF3h interaction. Mol Cancer. 2022;21(1):49. 35164758 10.1186/s12943-021-01471-y PMC8842935

[253] Zhou H , Huang X , Yang X , et al. CircRAPGEF5 promotes the proliferation and metastasis of lung adenocarcinoma through the miR‐1236‐3p/ZEB1 axis and serves as a potential biomarker. Int J Biol Sci. 2022;18(5):2116. 35342341 10.7150/ijbs.66770 PMC8935214

[254] Yang X , Xia J , Peng C , et al. Expression of plasma exosomal circLPAR1 in patients with gastric cancer and its clinical application value. Am J Cancer Res. 2023;13(9):4269. 37818058 PMC10560946

[255] Wei Y , Fu J , Zhang H , et al. N6‐methyladenosine modification promotes hepatocarcinogenesis through circ‐CDYL‐enriched and EpCAM‐positive liver tumor‐initiating exosomes. Iscience. 2023;26(10). 10.1016/j.isci.2023.108022 PMC10638478 37954137

[256] Hussen BM , Abdullah ST , Abdullah SR , et al. Exosomal non‐coding RNAs: blueprint in colorectal cancer metastasis and therapeutic targets. Noncoding RNA Res. 2023;8(4):615‐632. 37767111 10.1016/j.ncrna.2023.09.001 PMC10520679

[257] Yue X , Lan F , Liu W . Serum exosomal circCCDC66 as a potential diagnostic and prognostic biomarker for pituitary adenomas. Front Oncol. 2023;13:1268778. 38098508 10.3389/fonc.2023.1268778 PMC10720038

[258] Li P , Xu Z , Liu T , et al. Circular RNA sequencing reveals serum exosome circular RNA panel for high‐grade astrocytoma diagnosis. Clin Chem. 2022;68(2):332‐343. 34942001 10.1093/clinchem/hvab254

[259] Tang X , Deng Z , Ding P , et al. A novel protein encoded by circHNRNPU promotes multiple myeloma progression by regulating the bone marrow microenvironment and alternative splicing. J Exp Clin Cancer Res. 2022;41(1):85. 35260179 10.1186/s13046-022-02276-7 PMC8903708

[260] Wen N , Peng D , Xiong X , et al. Cholangiocarcinoma combined with biliary obstruction: an exosomal circRNA signature for diagnosis and early recurrence monitoring. Signal Transduct Target Ther. 2024;9(1):107. 38697972 10.1038/s41392-024-01814-3 PMC11636852

[261] Guo X , Gao C , Yang D‐H , et al. Exosomal circular RNAs: a chief culprit in cancer chemotherapy resistance. Drug Resist Update. 2023;67:100937. 10.1016/j.drup.2023.100937 36753923

[262] Zhang Z , Li X , Wang Y , et al. Involvement of inflammasomes in tumor microenvironment and tumor therapies. J Hematol Oncol. 2023;16(1):24. 36932407 10.1186/s13045-023-01407-7 PMC10022228

[263] Shang Z , Luo Z , Wang Y , et al. CircHIPK3 contributes to cisplatin resistance in gastric cancer by blocking autophagy‐dependent ferroptosis. J Cell Physiol. 2023;238(10):2407‐2424. 37566605 10.1002/jcp.31093

[264] Ma J , Chen C , Fan Z , et al. CircEGFR reduces the sensitivity of pirarubicin and regulates the malignant progression of triple‐negative breast cancer via the miR‐1299/EGFR axis. Int J Biol Macromol. 2023;244:125295. 37302631 10.1016/j.ijbiomac.2023.125295

[265] Gao J , Ao Y‐Q , Zhang L‐X , et al. Exosomal circZNF451 restrains anti‐PD1 treatment in lung adenocarcinoma via polarizing macrophages by complexing with TRIM56 and FXR1. J Exp Clin Cancer Res. 2022;41(1):295. 36209117 10.1186/s13046-022-02505-z PMC9547453

[266] Xu Y , Han J , Zhang X , et al. Exosomal circRNAs in gastrointestinal cancer: role in occurrence, development, diagnosis and clinical application. Oncol Rep. 2024;51(2):1‐14. 38099408 10.3892/or.2023.8678 PMC10777447

[267] Tang C , He X , Jia L , et al. Circular RNAs in glioma: molecular functions and pathological implications. Noncoding RNA Res. 2023;9(1):105‐115. 38075205 10.1016/j.ncrna.2023.10.007 PMC10700123

[268] Li Y , Hu J , Wang M , et al. Exosomal circPABPC1 promotes colorectal cancer liver metastases by regulating HMGA2 in the nucleus and BMP4/ADAM19 in the cytoplasm. Cell Death Discov. 2022;8(1):335. 35871166 10.1038/s41420-022-01124-z PMC9308786

[269] Chen C , Yu H , Han F , et al. Tumor‐suppressive circRHOBTB3 is excreted out of cells via exosome to sustain colorectal cancer cell fitness. Mol Cancer. 2022;21(1):46. 35148775 10.1186/s12943-022-01511-1 PMC8832727

[270] Barile L , Vassalli G . Exosomes: therapy delivery tools and biomarkers of diseases. Pharmacol Therapeut. 2017;174:63‐78. 10.1016/j.pharmthera.2017.02.020 28202367

[271] Lu Q , Kou D , Lou S , et al. Nanoparticles in tumor microenvironment remodeling and cancer immunotherapy. J Hematol Oncol. 2024;17(1):16. 38566199 10.1186/s13045-024-01535-8 PMC10986145

[272] Liao W , Du Y , Zhang C , et al. Exosomes: the next generation of endogenous nanomaterials for advanced drug delivery and therapy. Acta Biomater. 2019;86:1‐14. 30597259 10.1016/j.actbio.2018.12.045

[273] Shu G , Lu X , Pan Y , et al. Exosomal circSPIRE1 mediates glycosylation of E‐cadherin to suppress metastasis of renal cell carcinoma. Oncogene. 2023;42(22):1802‐1820. 37046045 10.1038/s41388-023-02678-7 PMC10238271

[274] Chen J , Wang H , Xu J , et al. CircZFR promotes colorectal cancer progression via stabilizing BCLAF1 and regulating the miR‐3127‐5p/RTKN2 axis. Sci China Life Sci. 2024:1‐18. 38805063 10.1007/s11427-023-2514-y

[275] Guo Z , Zhang Y , Xu W , et al. Engineered exosome‐mediated delivery of circDIDO1 inhibits gastric cancer progression via regulation of MiR‐1307‐3p/SOCS2 axis. J Transl Med. 2022;20(1):326. 35864511 10.1186/s12967-022-03527-z PMC9306104

[276] Geng X , Zhang Y , Lin X , et al. Exosomal circWDR62 promotes temozolomide resistance and malignant progression through regulation of the miR‐370‐3p/MGMT axis in glioma. Cell Death Dis. 2022;13(7):596. 35817771 10.1038/s41419-022-05056-5 PMC9273787

[277] Hao X , Zhang Y , Shi X , et al. CircPAK1 promotes the progression of hepatocellular carcinoma via modulation of YAP nucleus localization by interacting with 14‐3‐3ζ. J Exp Clin Cancer Res. 2022;41(1):281. 36131287 10.1186/s13046-022-02494-z PMC9494907

[278] Ye D , Gong M , Deng Y , et al. Roles and clinical application of exosomal circRNAs in the diagnosis and treatment of malignant tumors. J Transl Med. 2022;20(1):161. 35382838 10.1186/s12967-022-03367-x PMC8981684

[279] Lyu K , Tang B , Huang B , et al. Exosomal circPVT1 promotes angiogenesis in laryngeal cancer by activating the Rap1b‐VEGFR2 signaling pathway. Carcinogenesis. 2024:bgae030. 10.1093/carcin/bgae030 38824399

[280] Liu X , Guo Q , Gao G , et al. Exosome‐transmitted circCABIN1 promotes temozolomide resistance in glioblastoma via sustaining ErbB downstream signaling. J Nanobiotechnol. 2023;21(1):45. 10.1186/s12951-023-01801-w PMC9906870 36755314

[281] Zhang X , Xu Y , Ma L , et al. Essential roles of exosome and circRNA_101093 on ferroptosis desensitization in lung adenocarcinoma. Cancer Commun. 2022;42(4):287‐313. 10.1002/cac2.12275 PMC9017758 35184419

[282] Zhang Z , Gao Z , Fang H , et al. Therapeutic importance and diagnostic function of circRNAs in urological cancers: from metastasis to drug resistance. Cancer Metast Rev. 2024:1‐22. 10.1007/s10555-023-10152-9 38252399

[283] Wang X , Wang G , Wu Z , et al. Exosomal circ‐PTPN22 and circ‐ADAMTS6 mark T cell exhaustion and neutrophil extracellular traps in Asian intrahepatic cholangiocarcinoma. Mol Ther Nucl Acids. 2023;31:151‐163. 10.1016/j.omtn.2022.12.012 PMC9841234 36700045

[284] Du A , Yang Q , Sun X , et al. Exosomal circRNA‐001264 promotes AML immunosuppression through induction of M2‐like macrophages and PD‐L1 overexpression. Int Immunopharmacol. 2023;124:110868. 37657244 10.1016/j.intimp.2023.110868

[285] Li Y , Jiang B , Zeng L , et al. Adipocyte‐derived exosomes promote the progression of triple‐negative breast cancer through circCRIM1‐dependent OGA activation. Environ Res. 2023;239:117266. 37775001 10.1016/j.envres.2023.117266

[286] Yuan Z , Xiong B , Liu L , et al. Exosomal circ_0037104 derived from Hu‐MSCs inhibits cholangiocarcinoma progression by sponging miR‐620 and targeting AFAP1. J Biochem Mol Toxicol. 2024;38(2):e23656. 38348717 10.1002/jbt.23656

[287] Nicot C . RNA‐seq reveals novel CircRNAs involved in breast cancer progression and patient therapy response. Mol Cancer. 2020;19(1):76. 32293455 10.1186/s12943-020-01198-2 PMC7157975

[288] Shi Z‐X , Chen Z‐C , Zhong J‐Y , et al. High‐throughput and high‐accuracy single‐cell RNA isoform analysis using PacBio circular consensus sequencing. Nat Commun. 2023;14(1):2631. 37149708 10.1038/s41467-023-38324-9 PMC10164132

[289] Hansen TB , Venø MT , Damgaard CK , et al. Comparison of circular RNA prediction tools. Nucleic Acids Res. 2016;44(6):e58‐e58. 26657634 10.1093/nar/gkv1458 PMC4824091

